# Development and *in vivo* validation of small interfering RNAs targeting NOX3 to prevent sensorineural hearing loss

**DOI:** 10.3389/fneur.2022.993017

**Published:** 2022-09-16

**Authors:** German Nacher-Soler, Antoine Marteyn, Natasha Barenzung, Stéphanie Sgroi, Karl-Heinz Krause, Pascal Senn, Francis Rousset

**Affiliations:** ^1^The Inner Ear and Olfaction Lab, Department of Pathology and Immunology, Faculty of Medicine, University of Geneva, Geneva, Switzerland; ^2^Department of Pathology and Immunology, Faculty of Medicine, University of Geneva, Geneva, Switzerland; ^3^Department of Clinical Neurosciences, Service of ORL and Head and Neck Surgery, University Hospital of Geneva, Geneva, Switzerland

**Keywords:** gene silencing, NADPH oxidase 3, NOX3, inner ear delivery, inner ear therapy, canalostomy, bullostomy, *in situ* hybridization

## Abstract

The reactive oxygen species (ROS)-generating enzyme NOX3 has recently been implicated in the pathophysiology of several acquired forms of sensorineural hearing loss, including cisplatin-, noise- and age-related hearing loss. NOX3 is highly and specifically expressed in the inner ear and therefore represents an attractive target for specific intervention aiming at otoprotection. Despite the strong rationale to inhibit NOX3, there is currently no specific pharmacological inhibitor available. Molecular therapy may represent a powerful alternative. In this study, we developed and tested a collection of small interfering (si) RNA constructs to establish a proof of concept of NOX3 inhibition through local delivery in the mouse inner ear. The inhibitory potential of 10 different siRNA constructs was first assessed in three different cells lines expressing the NOX3 complex. Efficacy of the most promising siRNA construct to knock-down NOX3 was then further assessed *in vivo*, comparing middle ear delivery and direct intracochlear delivery through the posterior semi-circular canal. While hearing was completely preserved through the intervention, a significant downregulation of NOX3 expression in the mouse inner ear and particularly in the spiral ganglion area at clinically relevant levels (>60%) was observed 48 h after treatment. In contrast to successful intracochlear delivery, middle ear administration of siRNA failed to significantly inhibit Nox3 mRNA expression. In conclusion, intracochlear delivery of NOX3-siRNAs induces a robust temporal NOX3 downregulation, which could be of relevance to prevent predictable acute insults such as cisplatin chemotherapy-mediated ototoxicity and other forms of acquired hearing loss, including post-prevention of noise-induced hearing loss immediately after trauma. Successful translation of our concept into an eventual clinical use in humans will depend on the development of atraumatic and efficient delivery routes into the cochlea without a risk to induce hearing loss through the intervention.

## Introduction

Acquired (i.e. non-congenital) sensorineural hearing loss (ASHL) is a frequent degenerative disorder contributing to cognitive decline, depression and social isolation (1–3). The most prevalent forms of acquired hearing loss are caused by noise trauma, ototoxic drugs and aging, the last one affecting over a third of the population at retirement age (4). In contrast to congenital hearing loss, which is mainly caused by monogenic mutations, acquired forms of hearing loss are difficult to diagnose and to treat due to a complex combination of environmental and genetic factors. Furthermore, in mammals, hearing loss is irreversible since the cochlear sensory epithelium, comprising the sensory hair cells (HC) and associated auditory neurons (SGN), lost its ability to regenerate. Hearing aids and cochlear implants alleviate the symptoms but are not a causal treatment of hearing loss. Cochlear gene therapy may offer exciting alternatives in the future. However, so far, gene therapies are developed against inherited and monogenic forms of hearing loss, while ASHL, although much more prevalent in the society, has largely been neglected in this emerging field. Consequently, the development of an efficient prophylaxis or even regenerative treatment against ASHL is still an unmet clinical need.

Recent evidence demonstrates that the auditory synapse is the most vulnerable element in the auditory sensory transduction chain (5), preceding the loss of hair cells and auditory neurons. The so-called auditory synaptopathy is morphologically characterized by post synaptic terminal swelling and disruption of the pre- and post-synaptic components as a consequence of glutamate excitotoxicity and oxidative stress. Growing evidence point at NOX3 as a relevant source of ROS (6) in this context. NADPH oxidases (NOX), a family of ROS-generating enzymes (7), are emerging as potential pharmacological targets in numerous pathologies involving oxidative stress or aberrant redox signaling (8). The NOX family consists of seven members (NOX1, NOX2, NOX3, NOX4, NOX5, DUOX1, and DUOX2) with distinct tissue expression. Interestingly, the NOX3 isoform is exclusively and highly expressed in the inner ear (6, 9). NOX3 is needed for normal vestibular development (10), while is also expressed in the cochlea without clearly identified physiological function (11, 12). There is ample evidence implicating NOX3 in pathological ROS formation, ultimately leading to hearing loss. For instance, the chemotherapeutic and ototoxic drug cisplatin strongly enhances NOX3 expression (12, 13) and ROS generation (9). We have also demonstrated that in an aging mouse model, NOX3 deficiency prevents from excitotoxic cochlear damage associated to cochlear aging, showing significant protection of hearing and cochlear morphology (14). Furthermore, NOX3-deficient mice exhibit significantly better hearing recovery following noise trauma compared to their wild-type (WT) littermates (15). Interestingly, contrasting with the initial hypothesis of a direct oxidative stress mediated damage, the consequences of NOX3 activity are more complex, involving specific redox signaling pathways. Indeed, NOX3 was demonstrated to regulate the expression of genes involved in the excitatory pathway in auditory neurons, and auditory neurons from NOX3-deficient mice were protected from glutamate excitotoxicity, deciphering key molecular mechanisms leading to ASHL (14). Together, growing evidence indicates that NOX3 is a common source of ROS in several forms of acquired sensorineural hearing loss, namely noise trauma, cisplatin and age-related hearing loss (12).

Therefore, there is a strong rationale in targeting NOX3 activity to protect or promote regeneration of the auditory synapse in order to prevent early stages of acquired sensorineural hearing loss. Several antioxidant drugs, such as ebselen, D-methionine or N-acetylcysteine have been tested in clinical trials for hearing loss (16), but as of today, no efficient drug treatment or prophylaxis is available (17), probably due to a lack of target specificity. In the absence of pharmacological inhibitors of NOX3, molecular targeting via gene or RNA therapy offers a promising alternative, allowing specific NOX3 inhibition upon single intervention. Small interfering (si) RNAs are 19-22-nucleotide single stranded RNAs with a complementary sequence to the target gene mRNA; complementation leads to mRNA degradation and translational silencing (18). The use of siRNA results in a potent but transient target gene silencing, which may be of interest to prevent acute hearing trauma (e.g., noise or cisplatin-induced).

In the present study, we have developed an *in vitro* screening assay to evaluate the potency of 10 siRNA sequences targeting the NOX3 mRNA. We have identified and characterized several particularly efficient siRNAs sequences capable of silencing NOX3 with an IC_50_ in the sub nM range. One sequence is of particular interest regarding clinical translation, since the key NOX3 mRNA target sequence is conserved between mouse, guinea pig and human, allowing for direct comparison between species. The ability of this sequence to silence NOX3 *in vivo* was further tested comparing middle and inner ear delivery routes in mice. Significant NOX3 silencing was observed 48 h following posterior semicircular canalostomy delivery without inducing hearing loss. However, middle ear delivery failed to reach its target and led to adverse effects on animal hearing. Based on the results previously obtained from NOX3 knockout mice (12, 14, 15), we hypothesize that mice receiving NOX3 siRNA will be at least partially protected from noise-induced hearing loss. However, translation of such a concept toward clinical application in humans will request important efforts to achieve efficient intracochlear siRNA delivery without inducing hearing loss (19–21).

## Materials and methods

### Cell culture

HEK cells and HeLa cells were cultured in DMEM medium supplemented with 10 FBS, 1 L-Glutamin, and 1 % Pen/Strep. CHO cells were cultured in DMEM-F12 medium supplemented with 10 FBS, 1 L-Glutamin, and 1 % Pen/Strep (all from Gibco). Medium was changed every 2–3 days and cells were passaged when they reached 80–90 % of confluency.

#### Construction of plasmid and lentiviral vector

Using the Gateway recombination cloning technology, we cloned the coding sequences of Nox3 (NM_198958.2) and the three subunits constituting the mouse NOX3 complex, meaning Cyba (NM_007806.3), Noxo1 (NM_027988.4) and Noxa1 (NM_172204.4), under the human UBI promoter. In order to establish cell lines expressing constitutively all subunits of NOX3 complex, each subunit gene was cloned under a different selectable marker (GFP, mCherry, blasticidin, and puromycin respectively) under the control of the PGK promoter (Supplementary Figures 1A–C). Final lentivector was produced by transient transfection of HEK cells with the generated lentivector plasmid pCWX-UBI-IDUA-PGK-BSD, the pCAG-VSVG envelope plasmid and the psPAX2 plasmid encoding gag/pol, following the CaPO4 method (22).

Following a similar procedure, lentivectors expressing the coding sequences of each subunit of the human NOX3 complex have been produced NOX3 (NM_015718.2), CYBA (NM_000101.3), NOXO1 (NM_144603.3) and NOXA1 (NM_006647.1).

#### Lentivector transduction

The day before transduction, cells were seeded in six well plates at a density of 100,000 cells per well. The day after, cells were transduced with a low Multiplicity of Infection (MOI) (NOX3 =1; MOI other subunits = 5); a medium (MOI NOX3 =5; MOI other subunits =10); or a high MOI (MOI NOX3 = 10; MOI other subunits = 20) (Supplementary Figure 1D). Mediums were changed 1 day after transduction. Cells were cultured for 5 days before starting antibiotic selection: 5 μg/mL of blasticidin and 7 μg/mL of puromycin (all InvivoGen) were added to the medium during 3 weeks. Culture medium was changed every 2–3 days.

#### Flow cytometry

After 3 weeks of antibiotics pressure to select NOXO1 and NOXA1 positive cells, cells also expressing both NOX3 and CYBA were sorted by FACSAria II cell sorter (BD) based on the production of GFP and mCherry respectively.

#### ROS measurement

The production of ROS was measured by water-soluble tetrazolium salt (WST-1) reduction assay. Cells (HEK, HeLa and CHO) were detached by trypsinization (5 min at 37°C), washed with HBSS (Gibco), counted and resuspended in HBSS at 2,500,000 cells/ml. Cells were seeded in 384 well plates at a density of 10,000 cells per well (40 μL). Three-hundred and eighty-four well transparent plates (Corning) were used for absorbance readout (WST-1).

Cells were incubated with Diphenyleneiodonium (DPI) (10 μM), an inhibitor of ROS production (negative control), and/or the PKC activator phorbol myristate acetate (PMA) (100 nM) (NADPH activator) in HBSS for 10 min before measurement. For ROS measurement, WST-1 reagent (1 mM) was employed. Final volume was 40 μl per well. WST-1 experiments were performed at 37°C with SpectraMax Paradigm (Molecular Devices). Absorbance was read every 3 min during 8 h at 440 nm and 600 nm.

#### NOX3 SiRNA transfection

Transfection of Nox3 siRNA was performed both on 24 well plates, to measure the downregulation of Nox3 at the RNA level by qPCR, and on 384 well plates, to validate the inhibitory effect of our siRNA on ROS production (Amplex Red and WST-1 assay). A list of siRNA sequences and position within Nox3 cDNA (NM_198958.2) is available in Table 1.

**Table 1:** Sequence of siRNAs and their relative position on Nox3 mouse cDNA.

**Position in NM_198958.2**	**Core sense strand sequence**
28	AAAGGAAUAACGUGUUGGA
30	AGGAAUAACGUGUUGGAGU
187	ACACGAGUUAUUCUGGGUU
248	UUAACUGCAUGCUAAUUCU
272	CUGUCAGUCGGAACUUCAU
378	CUACGGGAUAGCUGUCAAU
503	CCCCAAAUGAGAGCUACCU
583	AUUACUGGCCUGGGUAUCU
735	CAUUCGAGGCCAAACUCCA
893	AUGCGUGUGAAAGAAUAAU

The day before siRNA transfection, cells were seeded at a density of 50.000 cells per well in 24 well plates, or 10.000 cells per well in 384 well plates. Before transfection, the culture medium was replaced by Opti-MEM^®^ I (Gibco). Lipofectamine™ RNAiMAX (Thermo Fisher Scientific) and siRNA were diluted separately in Opti-MEM^®^-I, according to manufacturer's instructions. The resulting siRNA mix and Lipofectime mix (both with Opti-MEM^®^-I) were posteriorly homogenized together, following manufacturer's instructions. 5 min after homogenization, the resulting solutions, containing independent siRNA sequences, were added to the cells into the different well plates. All NOX3 siRNA constructs were provided by Decibel Therapeutics. Fluorescent siGlo Green (Dharmacon) was used as a negative control siRNA.

#### Cochlear explant isolation, culture and transfection

Cochlear explants were isolated from P5 C57Bl/6J mice and were plated on coverslips coated with Matrigel and covered with DMEM/F12 medium containing N2 and B27 supplements (1X), 10% of fetal bovine serum (FBS) and Ampicillin. siRNA transfection was performed using Lipofectamine RNAiMAX, as soon as they attached to coverslips (around 1 h after plating). Cochlear explants were transfected either with increasing siGLO concentrations (20–80 nM), or siNOX3 (80 nM). At day 2 following isolation and transfection, mRNA was extracted from cochlear explant for qPCR analysis.

#### RNA extraction and RT-PCR on cell lines and cochlear explants

Total RNA was extracted according to the RNeasy Mini kit protocol (Qiagen). Then, reverse transcription was performed using the PrimeScript RT Reagent Kit (Takara). Finally, real-time polymerase chain reaction (RT-PCR) was achieved with PowerUp SYBR Green Master Mix (Applied biosystems) using the QuantStudio 12 K Flex Real-Time PCR System (Thermo Fisher Scientific). The efficiency of each primer was verified with serial dilutions of cDNA. Relative expression levels were calculated by normalization to the geometric mean of the three house-keeping genes (*Eef1a, Tubb* and *Actb* for mouse, *ACTB, B2M* and *GAPDH* for human and *Actb, Gapdh, Tubb* for chinese hamster (CHO cells) samples). Sequences of the primers used are available in Supplementary Table 1.

#### *In vivo* procedures

Fifteen Young Adult (7–8 Weeks old) C57BL/6, Male and Female Mice, Were Used During the *in Vivo* SiRNA Characterization. All *in Vivo* Experimental Protocols Were Approved by the Local Veterinary Office and the Commission for Animal Experimentation of the Geneva Canton, Switzerland, Authorization Number GE/149/18. Each Animal Underwent Canalostomy or Bullostomy Surgery Only Once. Animals Were Distributed in 4 Different Experimental Groups, Including SiNOX3 (*n* = 2) or SiGLO (*n* = 2) Delivered Through Bullostomy and SiNOX3 (*n* = 9) or SiGLO (n=2) Delivered Through Canalostomy. All Contralateral Ears Were Indistinctly Classified as non-Operated.

Animals were anesthetized by intraperitoneal injection (IP) of 10 Ketamine and 5% of Xylazine (dose of 10 μL/g) for all surgical procedures, auditory testing and immediately before sacrifice. During all surgical procedures, pedal reflex was monitored periodically (every 20 min), in order to confirm proper anesthesia depth. If required, a 10 % ketamine solution was injected intramuscularly (dose 5 μL/g) to elongate the surgical window. All animal subjected to surgery were provided with proper local analgesia (dose 1.2 μL/g), employing a lidocaine and epinephrine solution (0.5 %), during and after the procedure. Animal condition was monitored daily with a welfare score sheet until the experiment termination on day 3 (D3). Additionally, food pellets were disposed on the cage floor to facilitate food accessibility after canalostomy delivery.

Following auditory evaluation at D3, all animals were euthanized by cervical dislocation under general anesthesia, followed by decapitation and dissection. Collected samples were employed for further histological analysis and RNAscope *in situ* hybridization.

#### Animal care score sheet

Animal welfare was assessed by the recording of a score sheet, as previously described (23). The score sheet was filled at time D0 to normalize the data, and then every day after the surgery until D3. Different variables as weight (general health indicator), piloerection, ataxia, wound condition, behavior signs (reaction to manipulation and social behavior) and equilibrium (relevant evaluation following canalostomy delivery) were evaluated, allowing accurate welfare evaluation. Upon canalostomy delivery, animal equilibrium was daily assessed with the tail suspension test. Rotation along the tail axis was classified as vestibular deficit.

The animal scoring system was designed following severity from zero (normal state) to three. If a level one was detected, it was monitored closely, in a level two analgesia was injected and closely observed, and finally level three animals were sacrificed following ethical guidelines.

#### SiRNA preparation for *in vivo* delivery

The siRNA construct and siGlo reagent, used as control, were pre-incubated with a cationic lipidic formulation to facilitate delivery though cellular membranes. siRNA (40 μM) or siGLO (20 μM, Dharmacon™ Green siGLO™) were diluted in an equal volume of OptiMEM (ThermoFisher, 31985062) and the resulting sample was further mixed with Lipofectamine RNAiMax (ThermoFisher, 13778100), according to the manufacturer instructions. The final suspension, containing 10 μM siRNA or 5 μM siGlo was delivered to the middle ear through bullostomy, or to the inner ear through canalostomy.

#### Auditory brainstem response measurements

Operated and contralateral ears were subjected to single ear auditory brainstem response (ABR) measurements (*n* = 8). Each ear was stimulated independently coupling the speaker with a custom-made ear adapter, having the contralateral ear as an internal control. ABRs recordings were performed in a soundproof chamber (IAC Acoustics, IL, United States), as previously described (14, 23). For the stimulus generation and recording of responses, a multifunction IO-Card (National Instruments, Austin, TX, United States) was employed. Sound pressure levels (SPL) were regulated with an attenuator and amplifier (Otoconsult, Frankfurt, Germany). Moreover, the sound pressure was calibrated online before each measurement with a microphone probe system (Bruel&Kjaer 4191) placed near the animals' ears. Recorded signals were amplified and bandpass filtered (80 dB; 0.2–3.0 kHz) using a filter/amplifier unit (Otoconsult, Frankfurt, Germany).

Animals were anesthetized following intraperitoneal injection and placed inside the soundproof chamber upon a heating pad controlling body temperature. Electrodes were placed subcutaneously on the forehead of the mouse (+), on the mastoid of the recorded ear (–) and a reference electrode on the back. ABRs were recorded following a click stimulus (100 μs signal, comprising all frequencies from 2 to 32 KHz) and 9 pure tone frequencies individually (2 to 32 KHz, with a resolution of two steps per octave, 3-ms signal). ABR wave pattern outcome was averaged over 128 stimulus repetitions, for click and for each pure tone measurement. For all frequencies, ABRs were analyzed from 0 to 80 dB SPL in five dB steps. The animal hearing threshold, last SPL with preserved wave pattern, was determined by visual analysis. Threshold shifts were determined as the difference between D3 and D0 measurements. Following the ABR measurement, mice were placed in a recovery cage and body temperature was regulated with a heating pad until full recovery.

#### Middle ear delivery of SiRNA through bullostomy

Bullostomy was performed as previously described (23). Only the right ear underwent surgery, maintaining the contralateral ear as an internal control. A Wild Heerbrug laparoscopy microscope was employed to perform all surgeries. Following general anesthesia, animals were place on a lateral position (right ear upwards) upon a heating pad covered with a sterile cover, to control body temperature during the surgical procedure.

The retroauricular area, located posteriorly to the pinna, was cleaned with 70 % alcohol and properly shaved, followed by topical sterilization with an iodine solution (Betadine^®^). A 1 cm incision was made over the specified area, revealing the cervical trapezius muscle ventrally-anteriorly to the great auricular nerve. After muscle retraction, the otic bulla was identified ventrally to the facial nerve and drilled employing a 25 G needle. This surgical access allowed the direct contact of the siRNA solution with the cochlear round window, without any significant hearing loss as previously demonstrated (23). A 20 μL GELoader tip (Eppendorf) was employed to deliver the siNOX3 (*n* = 2) or siGLO (*n* = 2) solution into the middle ear through the bulla opening. During 15 min, the bulla was refilled eight times, with 2 μL each time, compensating the Eustachian tube drainage, and following every refill, the solution overflow was removed with a sterile surgical sponge (FST, 18105-03). After the 15 min delivery protocol, the bulla was rinsed three times with a 0.9 % saline solution and all liquid was removed from the surgical area and middle ear with a sterile surgical sponge. A piece of tissue was disposed over the bulla opening and sealed employing cyanoacrylate glue. Finally, the surgical opening was closed with a skin suture and lidocaine local analgesia was provided. Operated animals rested upon the heating pad until full recovery.

#### Inner ear delivery of SiRNA through canalostomy

Direct inner ear delivery through canalostomy was performed as previously described (24). The surgery was performed on the right ear, keeping the contralateral ear as an internal hearing and histological control (siNOX3 *n* = 6, siGLO *n* = 2). A Wild Heerbrug laparoscopy microscope was used to conduct all surgeries. Animals received general anesthesia by intraperitoneal injection of Ketamine and Xylazine, and the surgical protocol started once determined the proper anesthesia depth (following the stapedial reflex). The operated animal was placed in a lateral position, exposing the right ear, upon a heating pad covered with a sterile cover.

As described above for the bullostomy protocol, the retroauricular area was carefully shaved and sterilized with an iodine solution (Betadine^®^). The incision area from the canalostomy slightly differs from the bullostomy, located more dorsally above the mouse ear pinna midline (Supplementary Figure 4). Following incision, skin retraction and fascia dissection, the great auricular nerve can be found on the ventral section of the surgical area (Supplementary Figure 4B). The posterior canal access requires microdissection of the cervical trapezius muscle. A small incision was made caudally to the upper section of the great auricular nerve, allowing the retraction of the muscle from the skull bone surface. After retraction, the posterior semicircular canal can be easily spotted on the skull surface (Supplementary Figures 4C,D) and carefully drilled employing a 25 G needle. The leaking perilymph was removed employing a sterile surgical sponge. Following the canal opening, the siRNA solution (10 μM) was delivered into the semicircular canal employing a custom-made microinjection tool, consisting of a 25 μL Hamilton's syringe coupled with a MicroLumen^®^ surgical tube (Inner diameter: 0.09652 mm; Outer diameter: 0.13208 mm), prefilled with mineral oil. The bone-tube interface was sealed with muscle tissue and cyanoacrylate to prevent reagent leakage. siRNA or siGlo suspension (1 μL) was slowly injected following manual compression of the syringe plunger during 2 min, reducing the risks of liquid overpressure and subsequent cochlear damages. After reagent delivery, the anesthetized animal was maintained in lateral position for another 10 min, before retrieving the cannulation tube, to facilitate siRNA diffusion and distribution through the inner ear. Finally, the microinjection device was removed, and the bony canal aperture was occluded with tissue and sealed with cyanoacrylate glue. Afterwards, the cervical trapezius muscle was retrieved to its initial position, and the animal skin was carefully sutured and sterilized with an iodine solution (Betadine^®^). The animal received local analgesia with lidocaine and rested upon the heating pad until full recovery.

#### Cochlea dissection and preparation

Following the final ABR measurement at day 3, mice were euthanized by cervical dislocation under anesthesia, followed by decapitation. The temporal bone dissection was performed rapidly to reduce post-mortem histological degradation. A sagittal cut of the skull was performed and both temporal bones were harvested. Inner ear isolation was carried out on a petri plate filled with cold PBS (1X, Gibco^®^), removing the bony auditory bulla and exposing the cochlea. During the microdissection, the integrity of the cochlea and middle ear bones were routinely checked, in order to discard mechanically damaged or misfolded cochleae which could generate misleading conclusions. Operated and contralateral ears were processed separately.

Cochleae intended for histological analysis were submerged in 4 % PFA overnight at room temperature. Fixed inner ears were decalcified with USEDECALC reagent (Medite commercial solution) under sonication for 2 days (Medite, Cat. No. 03-3300-00), according to the manufacturer protocol. Cochleae intended for RNA quantification were quickly dissected in cold PBS, carefully removing blood, muscular and connective tissue, and immediately frozen on liquid nitrogen. Samples were stored at −80°C until RNA extraction.

#### Cochlear histology

Decalcified cochleae were microdissected under a binocular microscope (Nikkon, Japan), carefully removing the cochlea decalcified bony surface (cytocochleogram). The resulting sensory epithelium, still attached to the auditory neurons, was divided in three sections, corresponding to the basal, medial and apical cochlear turn (high to low frequencies respectively) and stored in PBS (1X) on a 96 well-plate at 4°C until immunostaining.

Decalcified samples headed for histological NOX3 expression assessment were sequentially dehydrated and embedded in paraffin, following a standardized protocol. Mid-modiolar cuts of 5 μm were processed, loaded onto gelatin-coated slides and stored at 4°C until immunochemistry or RNAscope (*in situ* hybridization) staining.

##### Immunofluorescence

Cytocochleogram cochlear samples were permeabilized (3 % Triton-X 100 in PBS 1X) for 30 min at room temperature. After rinsing with PBS (three times), tissue was incubated with the blocking solution (2% BSA, 0.01% Triton-X 100, in PBS) for 30 min and then incubated with the primary antibodies, recognizing the Myosin-7a (1:200 in blocking solution) (polyclonal rabbit anti Myosin-7a, Proteus) and β-3 tubulin (1:500 in blocking solution) (monoclonal mouse anti TUBB3, Biolegend) epitopes. Primary antibodies were incubated overnight at 4°C, with a 10 rpm agitation (gyratory rocker, Stuart SSM3). The following day, samples were washed 3 times with PBS and incubated with the secondary antibodies conjugated with Alexa 555 (donkey anti-rabbit, Life technologies) and Alexa 647 (donkey anti-mouse, Life technologies) (1:500 in blocking solution) for 2 h at room temperature, with a 10 rpm agitation. Finally, samples were rinsed with PBS (three times), placed on glass slides and mounted with Fluoroshield commercial media, containing 4',6-diamidino-2-phenylindole (DAPI) (Sigma).

Image acquisition was performed on a confocal microscope set-up, consisting of a Zeiss laser-scanning confocal microscope (Zeiss LSM700) and a CCD camera recording system (Leica Microsystems). Confocal 10 μm multi z-stack images (0.7 μm steps) were recorded from the sensory epithelium employing a Plan-Neofluar 20X/0.50 objective, and further processed and analyzed with the open-source software FIJI (ImageJ).

##### RNAscope^®^ preparation

Mid-modiolar slices headed for NOX3 *in situ* hybridization (*n* = 3) were loaded onto glass slides (Thermo Superfrost Plus^®^) and followed the manufacturer's RNAscope 2.5 HD Assay – BROWN assay (Bio-techne, Cat. No. 322310) protocol, as previously described (14). Paraffin sections (5 μm) were sorted in three different groups, corresponding with the negative control (DapB-C1, Bio-techne, Cat. No. 310043), positive control (Ppib-C1, Bio-techne, Cat. No. 313911) and NOX3 (Bio-techne, Cat. No. 481989) RNAscope probes. Selected mid-modiolar preparations were incubated with the assigned probe for 2 h at 40°C and posteriorly revealed with the provided detection kit (enzymatic immunohistochemistry). To facilitate cochlear structure recognition, Mayer hematoxylin staining (30 s) was used as counterstaining agent. Following the staining phase, slides were dehydrated, cleared and mounted with Tissue-Tek^®^ Glas™ mounting media (Sakura, Cat. No. 1408).

Image acquisition was performed on a widefield microscope set-up, consisting of a motorized Zeiss microscope (Zeiss Axio Imager M2) and an Axiocam 702 mono (Zeiss) recording system. Widefield 4.8 μm multi z-stack mid-modiolar images (0.6 μm steps) were recorded employing a 40X/1.4 EC Plan-Apochromat (oil) objective. In order to cover the entire Rosenthal's canal, each image was composed by four tiles (2 X 2), automatically recorded employing the motorized microscope function. Resulting files were further processed employing Zeiss commercial software and the open-source software FIJI.

##### Histological quantification (ImageJ)

Images obtained from cochlear immunofluorescence and mid-modiolar *in situ* hybridization were analyzed with the open-source software FIJI.

Confocal 10 μm multi-stack files, resulting from cytocochleogram image acquisition, were projected into a single plane reconstruction (2D) to facilitate analysis. Resulting 2D images were further processed to reduce background noise, employing the rolling ball algorithm (25) included on Image J, and adjust image clarity. Basal, medial and apical turns of operated and contralateral ears were visually examined for hair cell integrity (Myosin-7a and DAPI markers co-staining) and siGLO migration (488 λ signal) to the sensory epithelium and spiral ganglia. Multichannel three-channels) artificial color and individual channel images were generated to facilitate visualization and with informative purposes.

In order to quantify RNAscope signal, we recorded multi z-stack and multi-tile mid-modiolar files. Each image was composed by four tiles (2 X 2), overlapped into a single wide-field image employing Zeiss commercial software. RNAscope signal quantification was automatized with a macro developed in our laboratory, to ensure equal image processing with a comparison purpose. The automatized tool was developed employing the built-on FIJI's macro software, together with the open-source software Ilastik (26) as segmentation tool. For image pre-processing, single tile z-stack images were projected into an individual plane and divided into two channels employing the function color deconvolution (H & DAB). First of all, the brown channel, corresponding to the DAB signal (RNAscope), was processed to subtract noise background and adjust image clarity (25). The processed channel LUT was then inverted (dark background and RNAscope bright signal) and duplicated. While one copy was kept with the original color LUT (brown, employed in signal quantification), the automatized macro applied an Amber LUT filter into the second copy to generate an artificial color image (fluorescent like) to facilitate visualization. Secondly, similarly to the DAB channel, the hematoxylin channel (purple) was processed for background subtraction (25). Composite images composed by the RNAscope original signal (brown) and hematoxylin counterstaining were generated during the macro automatic process.

For the quantification, the automated tool loaded the hematoxylin channel and ask the user to manually determine the Rosenthal's area. The selected area was measured and displaced to the DAB channel (signal channel) of the corresponding sample, measuring the signal intensity inside the delimited section. This approach allows the RNAscope signal measurement restricted to the Rosenthal's canal, limiting off-target signal quantification. The resulting measure (A.U.) was normalized with the area size (A.U./mm^2^) and compared between the positive, negative and NOX3 (siRNA treated vs. untreated) RNAscope groups.

#### Cochlea RNA extraction and QPCR quantification

Cochleae intended for RNA extraction (*n* = 6 mice, 12 cochleae) were quickly dissected in cold PBS, with special emphasis on removing blood and surrounding tissue. Following dissection, the samples were immediately frozen in liquid nitrogen and kept at −80°C for posterior RNA extraction, as previously described (14). RNA extraction was performed following an standardized Qiagen RNeasy Micro kit protocol (27), adapting the lysis and homogenization steps. Sterilized steel beads and the tissueLyser buffer provided on Qiagen kit were employed for mechanical homogenization for 30 seconds at 30 rpm. The resulting lysate was then collected and 750 μL of trizol were added, inducing DNA and RNA precipitation, followed by the addition of 150 μL of chloroform. The mixture was mechanically homogenized (intense shaking) and centrifugated, collecting the aqueous phase. Finally, the collected phase followed the rest of the purification protocol described on the commercial kit.

Takara PrimeScript RT reagent Kit was employed for RNA purification and tritation. Following manufacturer's protocol, 500 ng of RNA were purified and prepared for cDNA synthesis. Real-time PCR was performed using SYBR green assay on a 7900HT SDS system from ABI, as previously documented (14). NOX1, NOX2, NOX4, Cyba and NOXO1 genes expression were normalized using Eef1a, Tubb and Actb house-keeping genes. Sequences of the primers used are available in the Supplementary Table 1. The highest normalized quantity was assigned a value of 1.0. Then, the fold change of each single interest gene was calculated from the quotient of means of the RNA normalized quantities and reported as ± SEM. Results were normalized to the contralateral (non-operated) ear, represented as 100 % gene expression.

#### Statistics

All data were analyzed using the GraphPad Prism software (version 9.2.0). Two-way ANOVA analysis, followed by Bonferroni multiple comparison correction, was applied during ABR threshold shift comparison. A non-parametric *t*-test (Mann-Whitney) was employed during each single gene qPCR gene comparison and during NOX3 RNAscope signal comparison between siNOX3 operated ears and contralateral ears (non-operated). RNAscope signal quantification was analyzed employing a two-way ANOVA with a Fisher's LSD test (each cochlear turn comparison stands alone, no multiple test correction required). Confidence interval, for all statistics, was set as 95 % (a = 0.05); ^*^, *p* < 0.05; ^**^, *p* < 0.01; ^***^, *p* < 0.005; ^****^, *p* < 0.0005.

## Results

### Generation of NOX3-expressing cell lines producing reactive oxygen species (ROS)

**Figure 1:**
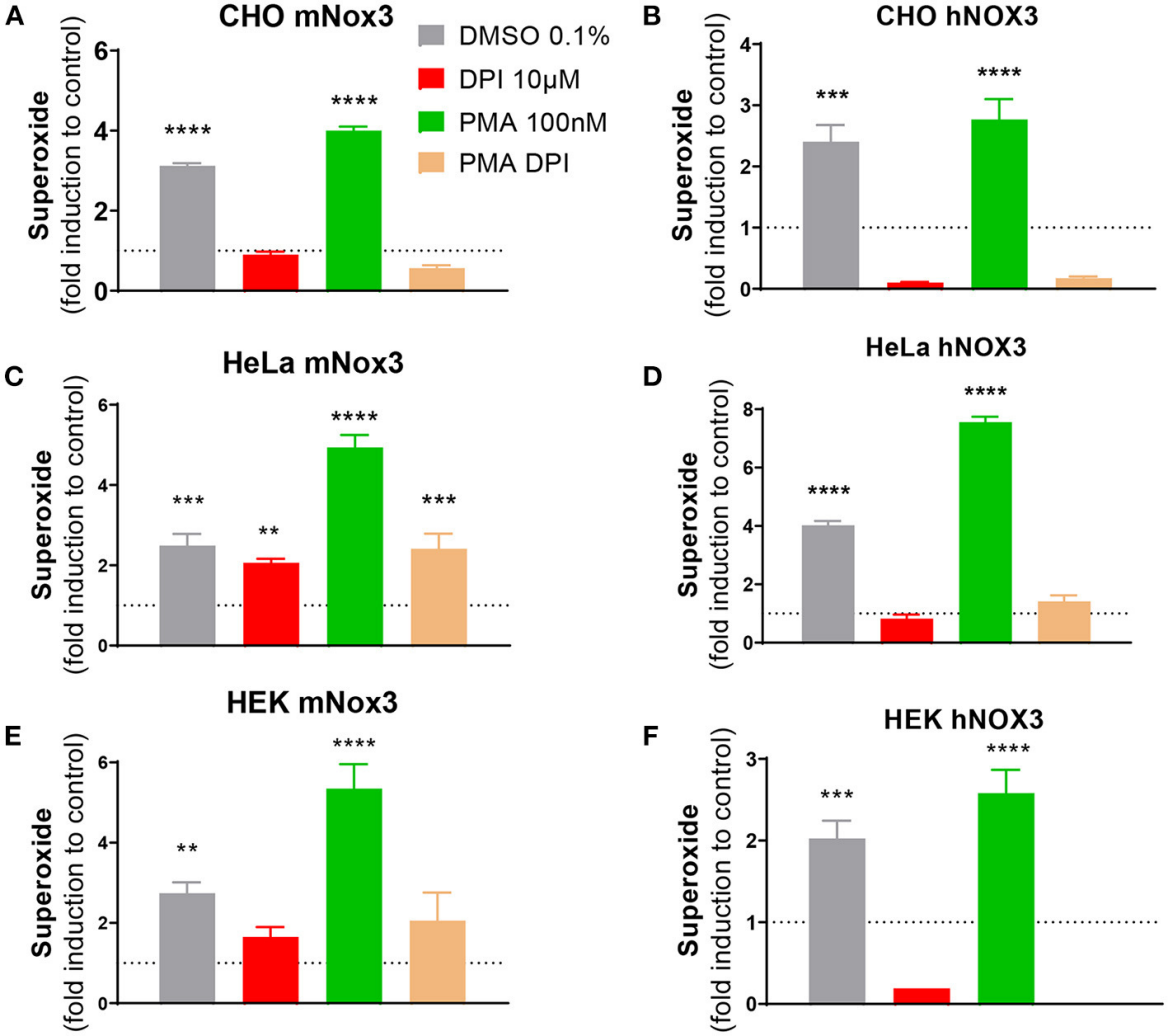
Characterization of NOX3 activity in three cell lines expressing the mouse or human NOX3 complex. ROS production was evaluated in **(A,B)** CHO, **(C,D)** HeLa and **(E,F)** HEK293 transduced with all subunits of **(A,C,E)** mouse (mNox3) or **(B,D,F)** human NOX3 complex (hNOX3) by using a colorimetric WST-1 assay, detecting superoxide. Bar graphs represent NADPH activity of NOX3 in cells treated with DMSO, the NOX3 agonist PMA and/or the NOX inhibitor DPI. Data from every experimental condition was compared and normalized to the untransduced control (WT) for each cell type. DMSO, dimethyl sulfoxide; PMA, phorbol 12-myristate 13-acetate; DPI, diphenyliodonium. Data represent the average of three independent experiment +- SEM.

In order to identify efficient NOX3 siRNAs, capable of inhibiting both, NOX3 expression and the resulting ROS production, we established cellular models expressing the four subunits of the mouse and human NOX3 complex (Figure 1). Namely, the coding sequence of NOX3, Cyba, NOXO1 and NOXA1 have been cloned under different selectable markers using the Gateway recombination cloning technology and have been integrated in HEK, HeLa and CHO cell line by lentivectors transduction. Cells expressing all subunits have been isolated and selected by cell sorting and antibiotic selection (Supplementary Figure 1). Each cell line has been generated with three different levels of multiplicity of infection (MOI): low, medium and high (Supplementary Figure 1). In all conditions, we chose to have the NOX3 subunit as the limiting subunit by transducing it with a lower MOI than other subunits to also observe effect of a silencing at the functional level.

At first, to validate our cellular models, gene expression of the different transduced subunits of the mouse NOX3 complex have been analyzed in the three generated cell lines. In all MOI conditions, the different subunits were relatively highly expressed (Ct value ≈ 25) compared to untransduced conditions which do not express any NOX3 subunit - or at a barely detectable level. We noticed that gene expressions were relatively similar between medium and high MOI conditions (Supplementary Figure 1D).

The production of ROS resulting from NOX3 activity has been tested by WST-1 in all generated conditions. We demonstrated that the NOX3 subunit had to be sufficiently expressed to induce a significant ROS production (>MOI 5). Indeed, cell lines generated with a low level of NOX3 (MOI =1) did not produce ROS above detection threshold. By contrast, cells transduced with medium and high MOI exhibited significant NOX3 activity when compared to untransduced cells (Figure 1; Supplementary Figure 1E). The addition of PMA, an activator of the protein kinase C promoting NOX3 complex activation, further increased NOX3-mediated ROS production in all cell lines (Figure 1; Supplementary Figure 1E). Interestingly, the effect of PMA was stronger in HeLa and HEK cell lines (~2.5-fold more) compared to the CHO cell line (<1.5-fold more). Oppositely, the addition of DPI (10 μM) completely abolished NOX3-dependent generation of ROS in all the cell lines. Note that in all generated cell lines every subunit of the NOX3 complex needed to be transduced for significant NADPH oxidase activity (CHO mNox3, Supplementary Figure 1E; other cell lines: not shown).

To exclude that a part of the ROS produced in our cell-lines was the result from other NOX isoforms (intrinsically expressed) interacting with the exogenous expression of Cyba, we generated similar cell lines but expressing a missense mutation (541: G/T → Asp/Tyr) in the transmembrane domain of NOX3, resulting in an absence of NADPH oxidase activity (Supplementary Figure 1E). Therefore, we developed and validated three cells lines expressing the mouse NOX3 complex and three cell lines expressing the human NOX3 complex, both able to produce NOX3-dependent NADPH oxidase activity, allowing to screen for inhibitors.

### Screening of siRNA able to knockdown NOX3 expression and activity in HEK NOX3-expressing cells

**Figure 2:**
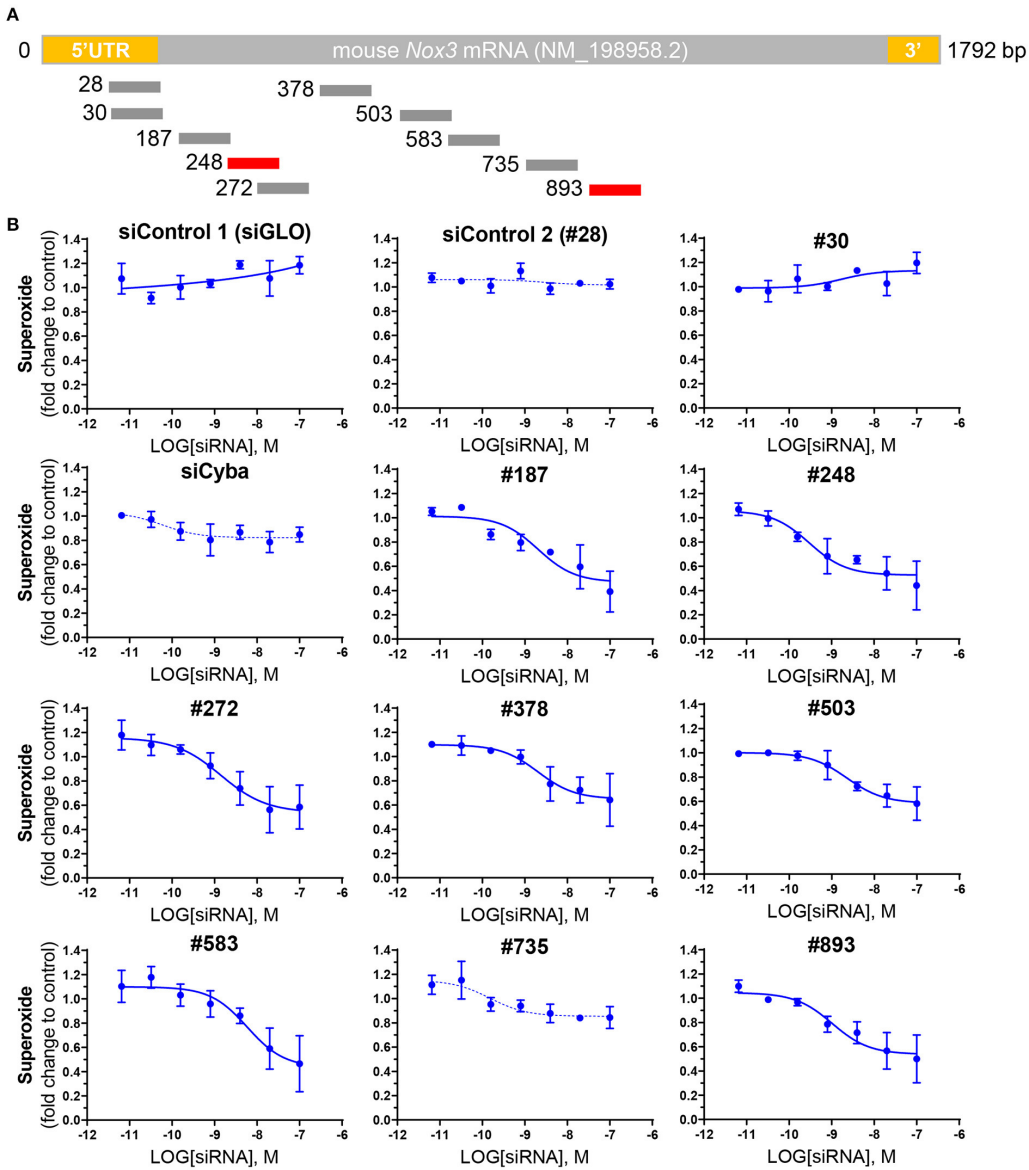
Screening of NOX3 siRNAs on mouse *Nox3* activity *in vitro*. **(A)** relative pairing region of the 10 siRNAs targeting the mouse *Nox3* mRNA on the mNox3 transcript. **(B)** ROS production measured by WST-1 in HEK cells expressing the mouse Nox3 complex (Nox3, Cyba, Noxa1, Noxo1; high MOI) transfected with increasing concentrations (0.0064 −100 nM) of the siRNAs of interest and control siRNAs [siGLO and siCyba]. siRNA concentrations are represented as the logarithm of the concentration (M). ROS production was normalized to the ROS values reported before siRNA treatment. Data represent the average of three independent experiment +- SEM.

In order to identify the most efficient NOX3 siRNA out of the library of 10 mouse NOX3 siRNA (Figure 2A; Table 1), we performed dose responses with seven increasing concentrations of siRNA (0.0064–100 nM) on our NOX3 expressing cells. 48 h later, half-maximal inhibitory concentration (IC_50_) was determined for each siRNA, based on the NADPH oxidase activity of NOX3 by WST-1 assay (Figure 2B). As expected, none of the two siRNA controls, namely irrelevant siRNA (siRNA negative control, siGLO) and siNOX3 28, which target the 5'UTR of NOX3, significantly affected NOX3 activity. By contrast, several mouse NOX3 siRNAs led to a significant NOX3 inhibition with an IC_50_ ranging from 0.2992 nM and 5.887 nM (Table 2). NOX3 siRNA 248 was identified as the most efficient construct with an IC_50_ of 0.2992 nM.

**Table 2:** IC_50_ of tested siRNAs on mNOX3 activity.

**# siRNA**	**IC_50_ (M)**
#187	2.030e-09
#248	2.992e-10
#272	1.399e-09
#378	2.035e-09
#503	2.265e-09
#583	5.887e-09
#893	1.034e-09

**Figure 3:**
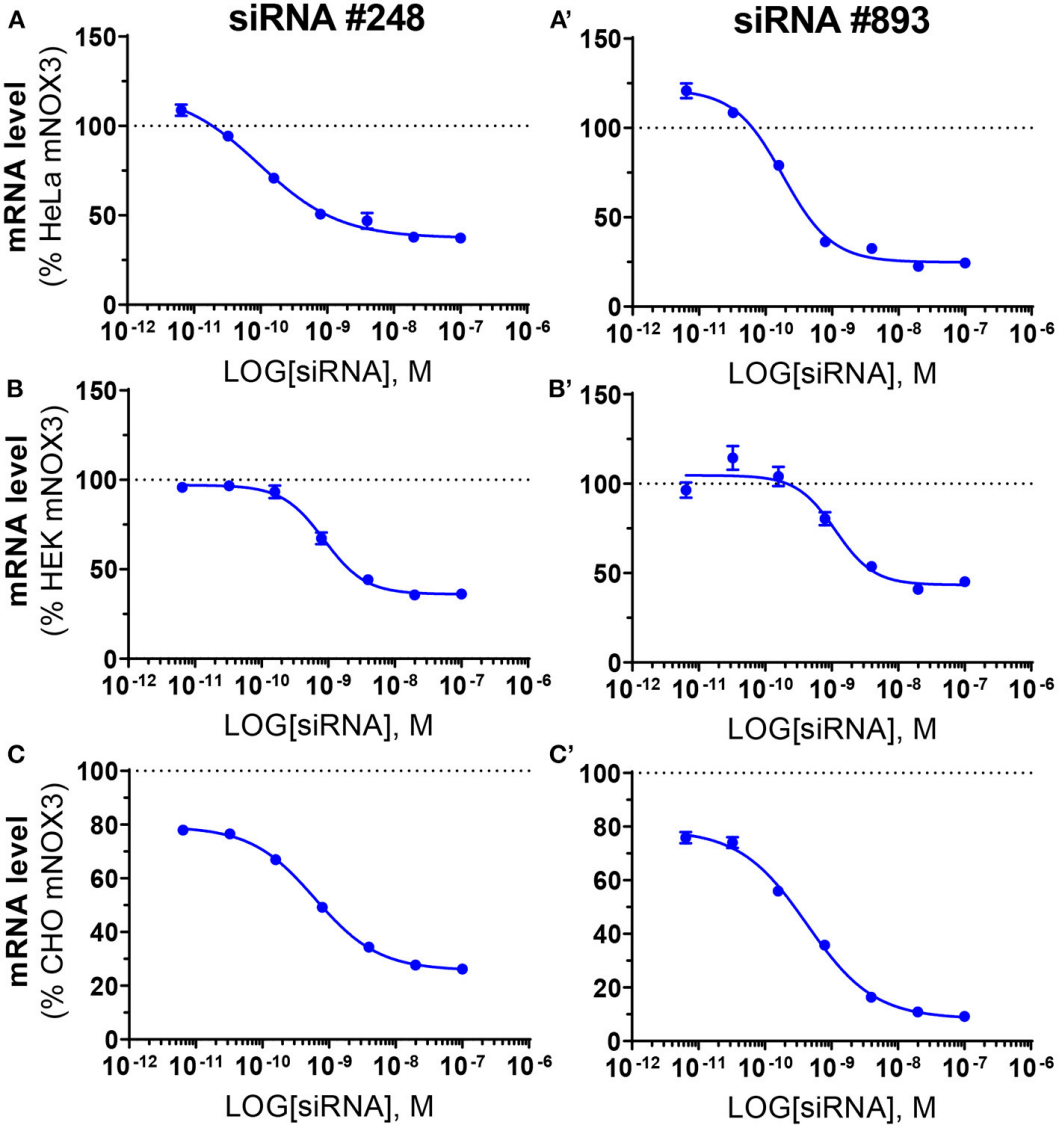
Validation of the efficiency of siRNA #248 and #893 on *Nox3* mRNA level in three *mNox3* expressing cell lines. NOX3 mRNA measured by qPCR in NOX3 expressing **(A)** HeLa, **(B)** HEK and **(C)** CHO transfected with increasing concentrations (100–0.0064 nM) of siRNA #248 **(A–C)** and #893 **(A'–C')**. siRNA concentrations are represented as the logarithm of the concentration (M). NOX3 mRNA expression was normalized to non-transfected cells. Data represent the average of three independent experiment +- SEM.

The two most potent NOX3 siRNAs (248 and 893) were also validated at the transcriptomic level by quantitative reverse transcriptase polymerase chain reaction (qRT-PCR) (Figure 3). Consistent with the WST-1 assay, siRNAs 248 and 893 led to a robust NOX3 mRNA silencing with an IC_50_ ranging between 0.1 nM and 1.13 nM in the three mouse cell lines (Table 3). By contrast, the expression level of other NOX3 subunits, namely *Cyba* and Noxo1 remained unaffected (Supplementary Figure 2).

**Table 3:** IC_50_ of siRNA #248 and #893 on Nox3 expression (mRNA) in the three mNox3 cell lines.

**IC_50_ (M)**	**#248**	**#893**
HEK	8.622e-10	1.129e-9
HeLa	9.630e-11	1.830e-10
CHO	6.124e-10	4.286e-10

### Silencing of NOX3 in cochlear explants

**Figure 4:**
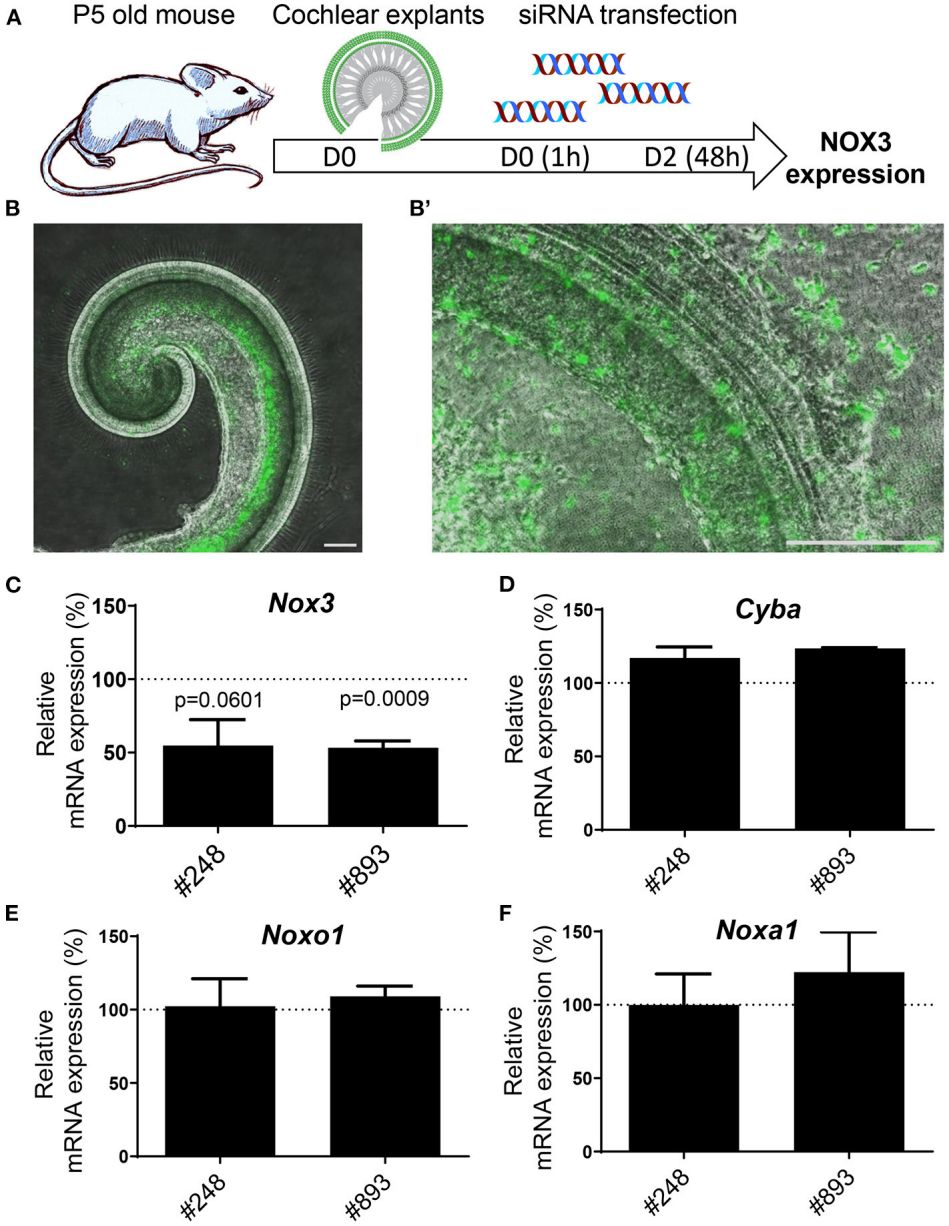
Efficiency of siRNA #248 and #893 on mouse cochlear explants. **(A)** Mouse cochlear explants were isolated from 5 days old C57Bl6/J mice and transfected with either fluorescent siRNA (siGLO) or Nox3 siRNAs #248 and #893**. (B)** Fluorescence microscopy picture of cochlear explants transfected with 80 nM siGLO (left panel). High magnification (right panel) revealed localization and cellular types of transfected cells. Data are representative from three independent experiments. Scale bars 200 μm. C-F) mRNA level of transcripts encoding for subunits of the NOX3 complex in transfected explants. The relative mRNA level is normalized to explants transfected with the control siRNA (100%). Data represent the average of three independent experiment +- SEM.

Mouse cochlear explants were transfected with increasing concentrations of fluorescent-labeled siGLO (previously used as siRNA negative control *in vitro*) (Figure 4A). At day two following siGLO transfection, fluorescence could be detected for concentrations of siRNA beyond 60 nM (not shown). However, based both on number of labeled cells and fluorescence intensity, we selected the concentration of 80 nM of siRNA as a reference for Nox3 siRNA transfection experiments (Figure 4B). Most of the transfected cells were neurons located in the helicotrema as well as some hair cells exhibiting fluorescence at high magnification (Figures 4B,B'). Transfection of *Nox3* siRNA #248 and #893 led to a decrease of *Nox3* expression by about 50% (Figure 4C) without changes in the expression of other subunits of the complex (Figures 4D–F).

### Anticipating translation of a SiRNA-mediated therapy for hearing loss

**Figure 5:**
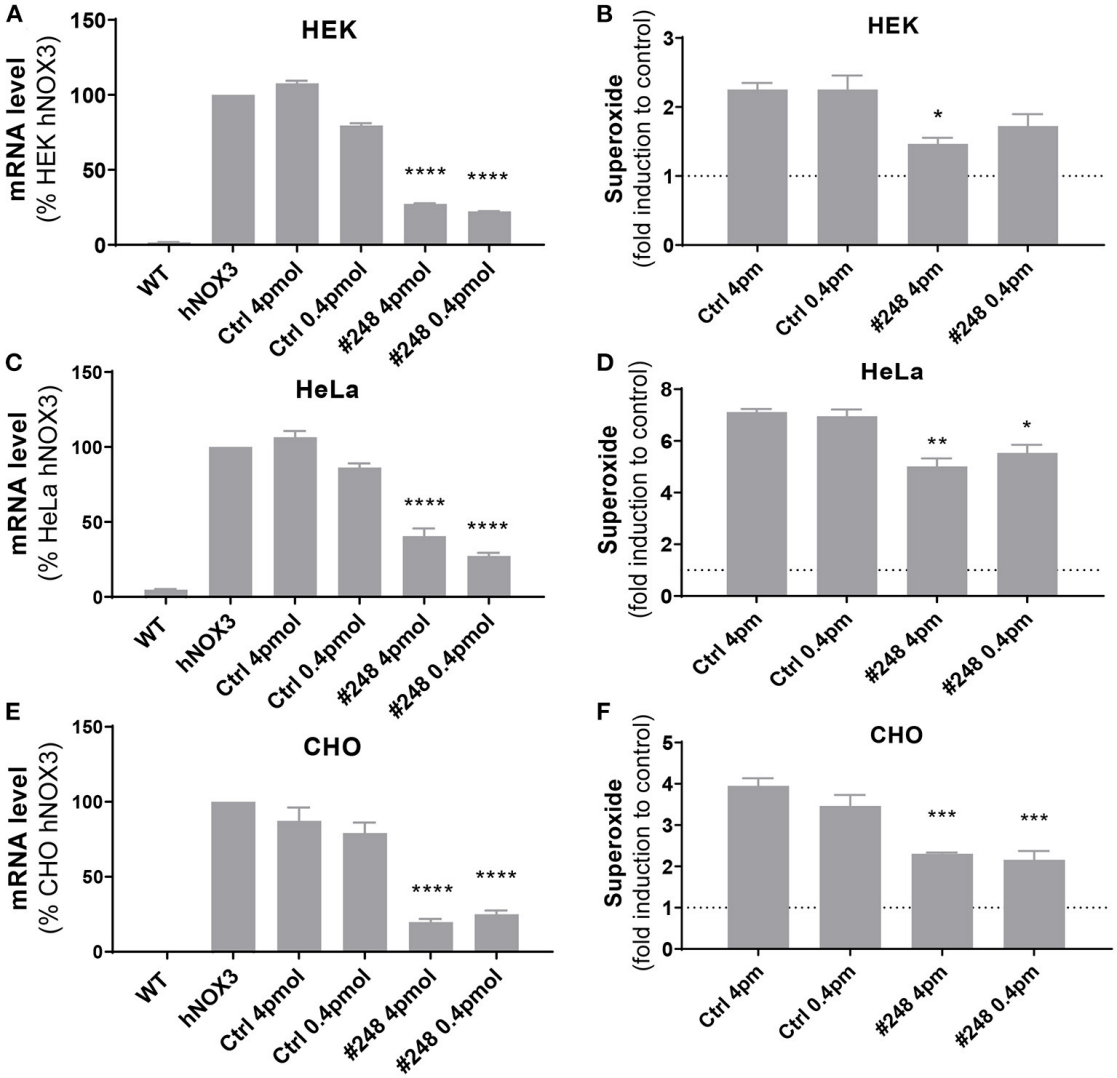
Validation of the efficiency of siRNA #248 on the human NOX3 complex. **(A,B)** HEK, **(C,D)** HeLa and **(E,F)** CHO cell lines expressing the human NOX3 complex (NOX3, CYBA, NOXO1 and NOXA1) were transfected with siRNA #248 or control siRNA (Ctrl) at two concentrations (4 and 0.4 pmol). **(A,C,E)** 48 h later, NOX3 mRNA was measured by qPCR in the three cell lines and **(B,D,F)** NADPH oxidase of NOX3 assessed using WST-1 assay. NOX3 mRNA expression was normalized to non-transfected hNOX3 expressing cells (100%). NOX3 activity was normalized to WT cells (1). Data represent the average of three independent experiments +- SEM.

Interestingly, the most potent NOX3 siRNA (#248) fully matched with the human NOX3 sequence and is therefore of interest for possible cross species comparison and eventual translation into human applications. Consequently, the silencing efficiency of this construct was assessed on the three generated human NOX3 cell lines (Figure 5). Efficacy and specificity of siRNA 248 has been assessed by its capacity to decrease Nox3 expression (qRT-PCR) and NADPH oxidase activity (WST-1). Upon human NOX3 siRNA treatment, siRNA 248 exhibited potent ROS inhibition and Nox3 mRNA silencing (Figure 5). No effect was detected on other subunits (Supplementary Figure 3). Therefore, based on its *in vitro* potency and its translatability, siRNA 248 was selected for further *in vivo* characterization in the mouse.

### Atraumatic SiRNA delivery to the mouse cochlea

**Figure 6:**
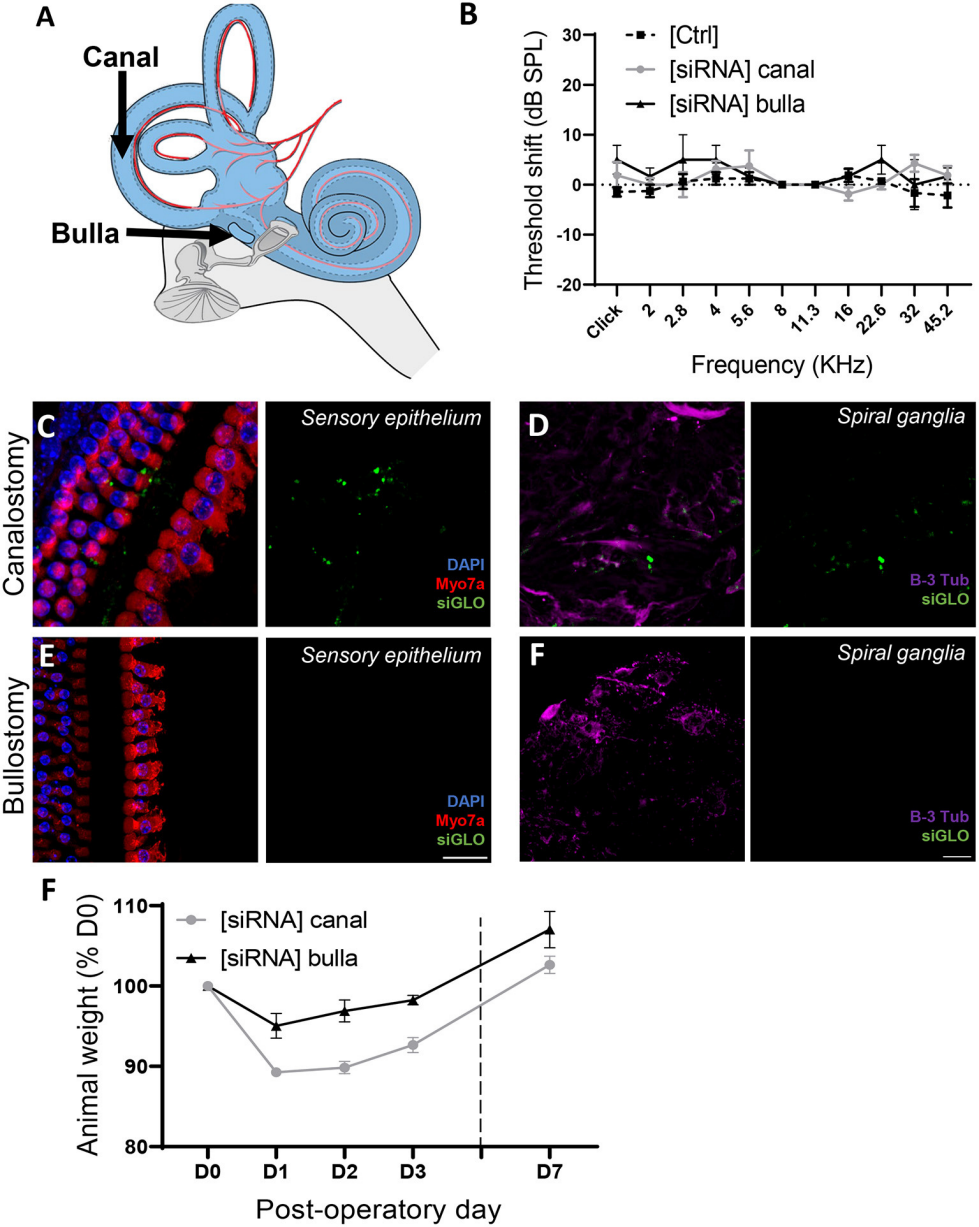
Atraumatic siRNA delivery of siRNAs to the mouse inner ear. **(A)** 1 μL of fluorescently conjugated siRNA (siGLO) was manually delivered into the middle ear, through the tympanic bulla (Bulla) or directly to the inner ear through the posterior semicircular canalostomy (Canal). **(B)** Auditory threshold shift measured by unilateral auditory brainstem response (ABR), recorded from contralateral [Ctrl] and operated [siRNA] ears (*n* = 12) 3 days after surgery. **(C,D)** Cytocochleograms showing siGLO diffusion (green) to the sensory epithelium (inner and outer hair cells) **(C)** and spiral ganglia (auditory neurons**) (D)** following canalostomy. **(E,F)** Cytocochleograms showing siGLO diffusion (green) to the sensory epithelium (inner and outer hair cells) **(E)** and spiral ganglia (auditory neurons) **(F)** following bullostomy. **(C,E)** Myo7a was used as hair cell marker (red), and DAPI (blue) was used for nuclear staining. **(D,F)** B-III Tubulin was used as neuron marker (purple). **(F)** Postsurgical weight evolution of operated animals was monitored up to up to day 7.

Inner ear delivery of therapeutics represents an important challenge nowadays (28, 29). The aforementioned, fluorescently conjugated siRNA (siGLO), was used to compare the middle ear and direct inner ear delivery in the mouse model *in vivo* (Figure 6A). The bullostomy protocol, allowing delivery to the middle ear, was based on a previous study (23), allowing the delivery of up to 16 μL of siRNA suspension, following eight repeated deliveries of 2 μL. The canalostomy procedure, based on a previous protocol (24), provides a direct access to the inner ear environment, allowing the direct delivery of up to 1 μL siRNA suspension into the cochlear/vestibular perilymph (Supplementary Figure 4). At day 3 following the siRNA delivery, neither the bullostomy nor the canalostomy procedure led to a significant elevation of hearing thresholds (Figure 6B). Following ABR hearing measurements, animals were sacrificed to evaluate the diffusion of the fluorescent siRNA along the tonotopic axis of the cochlea. Despite the higher amount of siRNA delivered through middle ear delivery, the intracochlear fluorescent signal was below detection threshold, indicating its inability to cross the round window membrane (RWM) (Figures 6E,F). By contrast, siGLO fluorescence was detected in the cochlear duct following canalostomy, including the organ of Corti (Figure 6C) and the spiral ganglion area (Figure 6D), demonstrating that a direct inner ear approach is required for efficient cochlear siRNA delivery. Note that the canalostomy procedure led to a transient vestibular dysfunction followed by rapid recovery in all animals (within 24 h), leading to a transient weight loss that was fully compensated by day 7 (Figure 6F).

### Efficient silencing of cochlear NOX3 following canalostomy-mediated SiRNA delivery

**Figure 7:**
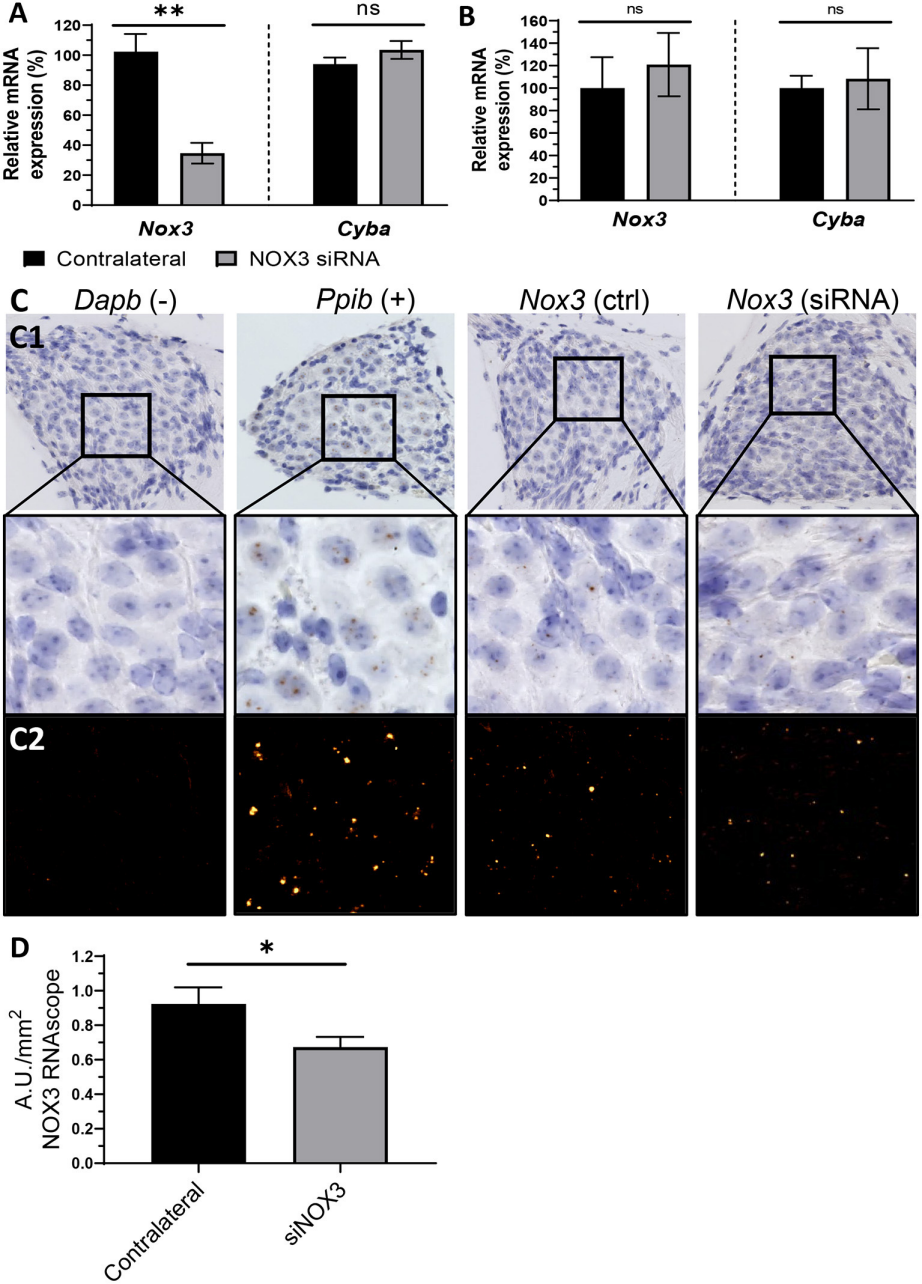
Silencing of NOX3 *in vivo*. NOX3 siRNA #248 was manually delivered to the inner ear through the posterior semicircular canal or into the middle ear through the tympanic bulla. **(A)** Bar graph showing the mRNA level (qPCR) of *Nox3* and p22^phox^ (*Cyba*) 3 days following inner ear delivery (canalostomy) (*n* = 6). **(B)** Bar graph showing the mRNA level (qPCR) of *Nox3* and p22^phox^ (*Cyba*) 3 days following middle ear delivery (*n* = 2). **(A,B)** mRNA level in the siRNA delivered cochlea was normalized to the contralateral cochlea (100%). The data represent the average +- SEM. **(C)** Representative images corresponding to the Rosenthal's canal *in situ* hybridization (BROWN assay) and hematoxylin counterstaining (C1). Dapb (dihydrodipicolinate reductase) probe was employed as negative control (–), while Ppib (Peptidyl-Prolyl Cis-Trans Isomerase was employed as positive control (+). DAB channel, corresponding to the mRNA signal, was processed in a fluorescent like manner (LUT applied), in order to facilitate visualization (C2). **(D)** Bar graph representing RNAscope (*in situ* hybridization) *Nox3* mRNA signal quantification in the Rosenthal's canal area. *Nox3* signal was normalized to the Rosenthal's area (mm^2^). The data represents the average +- SEM of *Nox3* signal quantified from five to eight cochlear slices / cochlea from three animals.

In order to provide quantitative and qualitative insights assessing the siNox3 silencing efficiency (#248), we performed RT-qPCR on the whole mouse cochlea as well as RNAscope *in situ* hybridization (Figure 7). The contralateral non-operated ear was used as an internal control. Consistent with the aforementioned fluorescence data, no significant Nox3 silencing effect of our siRNA construct could be measured 3 days following middle ear delivery (Figure 7B). However, following canalostomy, our data showed significant Nox3 mRNA knockdown (≈70 % at day 3 post-delivery) (Figure 7A). Knockdown was restricted to the genetic target (*Nox3*), since no effect was observed on other isoforms from the NOX family or subunits (*Nox1, 2* and *4*, Noxo1 and *Cyba*) (Figure 7A; Supplementary Figure 5) demonstrating the efficacy and specificity of our approach. We have previously demonstrated that the spiral ganglion is the primary location for Nox3 expression (14). In order to evaluate the impact of siRNA #248 on cochlear expression of Nox3, we performed RNAscope *in situ* hybridization on the operated mouse cochleae (Figure 7C). Significant expression of Nox3 was detected in the spiral ganglion area (Figure 7C), however not in the organ of Corti and in the stria vascularis area (not shown). By contrast, the Peptidylprolyl Isomerase B (PPIB) transcript, used as positive control was found to be expressed in any part of the cochlea, whereas no signal could be detected for the negative control, dihydrodipicolinate reductase (Dapb, bacterial gene). Importantly, following #248 siRNA delivery, we observed a significant *Nox3* mRNA silencing in the spiral ganglion neurons (A.U./mm^2^) when compared to the contralateral non-operated cochleae (ctrl) (Figures 7C,D). Together, the data demonstrate that canalostomy allows significant siRNA mediated silencing of our intracochlear target gene, namely Nox3. By contrast, when delivered to the middle ear, the siRNA failed to reach the cochlea.

## Discussion

The present experiments provide evidence for a potent siRNA-mediated knockdown of intracochlear NOX3 activity in mice. Silencing was achieved with a highly potent and translatable siRNA sequence, conserved between mouse, guinea pig and human, which may be of relevance for eventual human translation in the future. All siRNA sequences used in the study were identified *in vitro* by using a newly developed screening platform, allowing to select siRNA sequences based on their potency to inhibit NOX3 at the expression and activity level.

Oxidative stress is associated with many forms of acquired hearing loss and antioxidants have therefore been considered as potential prophylactic drugs particularly in the context of noise-induced hearing loss (30, 31). Despite overall promising preclinical data, many trials have been abandoned due to a lack of efficacy in humans (31). Antioxidants are likely to counteract the acute phase of ROS production leading to oxidative stress- mediated unspecific damages. However, specific redox signaling, in particular mediated by NOX3 activity as a key player of sensorineural damage emerges as a novel paradigm (14). While oxidative stress-mediated damages are largely unspecific, NOX-derived redox signaling is fined tuned by mechanisms including subcellular compartmentation, fine-tuned regulation of NOX activation and induction of antioxidant enzyme expression at close proximity to the source of ROS (32). Considering also the high reactivity and therefore extremely short half-life of many ROS, the overall disappointing efficacy for extrinsic antioxidants to inhibit specific redox pathways is not surprising. Yet, targeting the enzymatic complex at the origin of pathogenic redox signaling – namely NOX3 – rather than scavenging its product, appears as a promising strategy in acquired hearing loss. Molecular strategies, including gene or RNA mediated silencing present the advantage of acting upstream of the target with high specificity and controlled off target effects.

Several previous studies have demonstrated the efficiency of siRNA to achieve target gene knockdown in the cochlea in animal models (33). Interestingly, direct middle ear delivery of siRNAs, either transtympanically (13), or through retro-auricular surgery (34), has led in some studies to a significant knockdown of the target gene in the cochlea. In the present study, however, siRNAs failed to silence NOX3 mRNA when delivered through the tympanic bulla, which in addition resulted in significant conductive hearing loss. These discrepancies are difficult to explain but may reside in intrinsic differences between protocols, such as the use of a transfection reagent, the concentration of siRNAs or the animal model. In our hands, a direct inner ear delivery was required for efficient Nox3 silencing. For this purpose, we chose the posterior semicircular canal route, which was demonstrated to be atraumatic for hearing (24). Indeed, potent knockdown could be observed following posterior semicircular canalostomy without any detectable adverse effects on hearing. This suggests that the round window membrane remains a major obstacle for siRNA penetration to the mouse cochlea. In our hands, a liposomal transfection reagent did not help siRNAs to cross the RWM and led to middle ear dysfunction and subsequent conductive hearing loss. Whether, other carriers with different biophysical properties, such as poly-lactic based carriers (35), may be useful at enhancing RWM diffusion of siRNAs remains to be addressed.

Our best siRNA sequence, able to silence NOX3 with high affinity, targets a region of the NOX3 gene highly conserved between mouse, guinea pig and human and is therefore of high interest for eventual translation. Before being considered, however, the atraumatic intracochlear delivery needs to be established also for human applications. We and others have effectively used cochlear delivery (cochleostomy) or vestibular delivery (canalostomy) (24, 36) in mouse. Although such a direct inner ear delivery is well tolerated in the mouse model, with little to no damage to hearing function (37), it carries the risk of irreversible damage to residual hearing in human patients and is therefore not acceptable today. To overcome this bottleneck, 3D printed microneedles (38) and other atraumatic solutions need to be carefully assessed in animal and eventually, human models.

An advantage of siRNA-based therapy is the fact that it does not require transgene genome or epigenome insertion and does not carry the risk for sustained side effects or oncogenic transformations (19, 39). siRNA-based therapies could therefore be of major interest in scenarios with acute hearing insults, including noise trauma or drug-induced-hearing loss (i.e., cisplatin, aminoglycosides...). Because siRNA mediated silencing effects are transient, siRNAs are conceptually not well suited to prevent long-lasting or repeated insults to the cochlea (e.g., such as occurring in age-related hearing loss). For chronic conditions, long-lasting solutions such as viral vector-mediated gene therapy are more appropriate but are still far from any clinical application for otoprotection or otoregeneration in the context of an acquired form of hearing loss (40).

In conclusion, molecular approaches, including siRNA therapies, offer promising new perspectives to prevent acquired forms of hearing loss and address this unmet clinical need. In the absence of pharmacological inhibitors of NOX3, molecular therapies may be of clinical relevance in the near future, as NOX3-deficiency has recently been shown to protect from acquired forms of hearing loss (13–15). Before translation to human applications, atraumatic intracochlear delivery must be developed meeting human-grade requirements.

## Data availability statement

The original contributions presented in the study are included in the article/Supplementary material, further inquiries can be directed to the corresponding author/s.

## Ethics statement

All *in vivo* experimental protocols were approved by the local Veterinary Office and the Commission for Animal Experimentation of the Geneva Canton, Switzerland, authorization number GE/149/18.

## Author contributions

PS, FR, and K-HK conceived the project. GN-S, AM, and FR conceived and planned the experiments, drafted the manuscript and designed the figures. AM and NB carried out the *in vitro* experiments and analysis. GN-S carried out the *in vivo* experiments. GN-S and SS performed cochlear histology and analysis. FR supervised data curation. PS finalized the manuscript writing. PS, FR, and K-HK provided funding support. All authors contributed to the article and approved the submitted version.

## Funding

The authors acknowledge financial support from Decibel Therapeutics, Fondation Bodiféé, Ligue Genevoise contre le cancer, Fondation Gertrude Von Meissner, Fondation Louis Jeantet, Fondation Auris and Fondation Barguf. Statement: Open access funding provided by University Of Geneva.

## Conflict of interest

The authors declare that this study received funding from Decibel Therapeutics. The funder was involved in the study design. However, the funder had no role in the data collection, analysis, interpretation, the writing of this article or the decision to submit it for publication.

## Publisher's note

All claims expressed in this article are solely those of the authors and do not necessarily represent those of their affiliated organizations, or those of the publisher, the editors and the reviewers. Any product that may be evaluated in this article, or claim that may be made by its manufacturer, is not guaranteed or endorsed by the publisher.
