# Supplementary material for: Development and in vivo validation of small interfering RNAs targeting NOX3 to prevent sensorineural hearing loss
Source: Front Neurol. 2022 Sep 16;13:993017. doi: 10.3389/fneur.2022.993017 (PMC9523672; doi:10.3389/fneur.2022.993017)
Supplement: Supplementary file 1 [file Presentation_1.pdf]

Sup. Figure 1

A

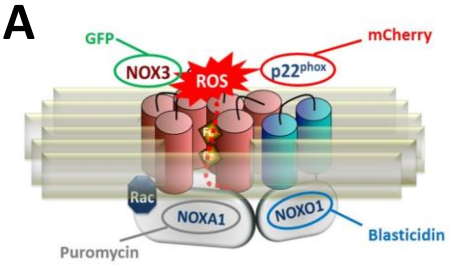

B

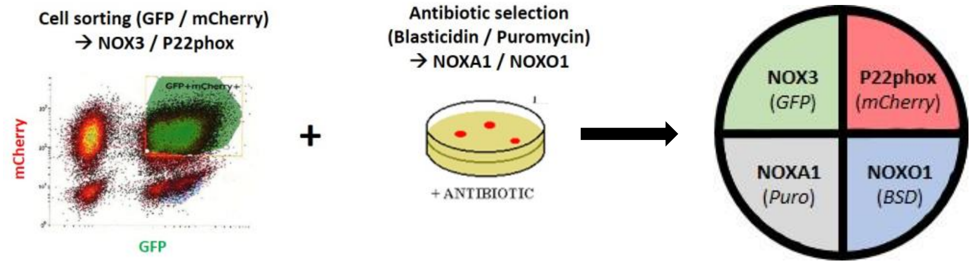

C

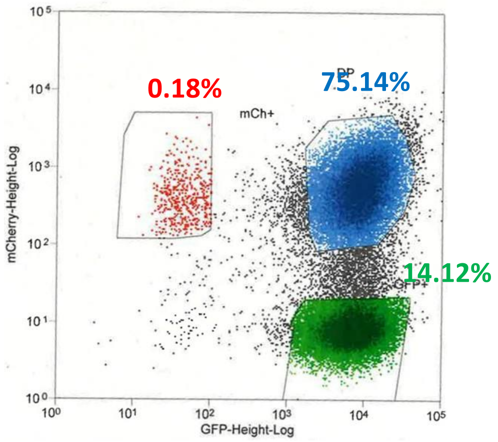

D

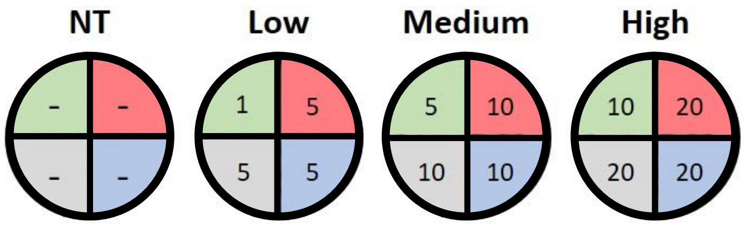

	Untransduced (NT)	Low (MOI=1)	Medium (MOI=5)	High (MOI=10)
GADPH	19.22	19.27	18.98	19.03
mNox3	-	27.67	24.96	24.74
mP22phox	-	24.48	23.85	23.87
mNoxo1	-	25.96	25.06	24.99
mNoxA1	-	26.96	26.29	26.10

E

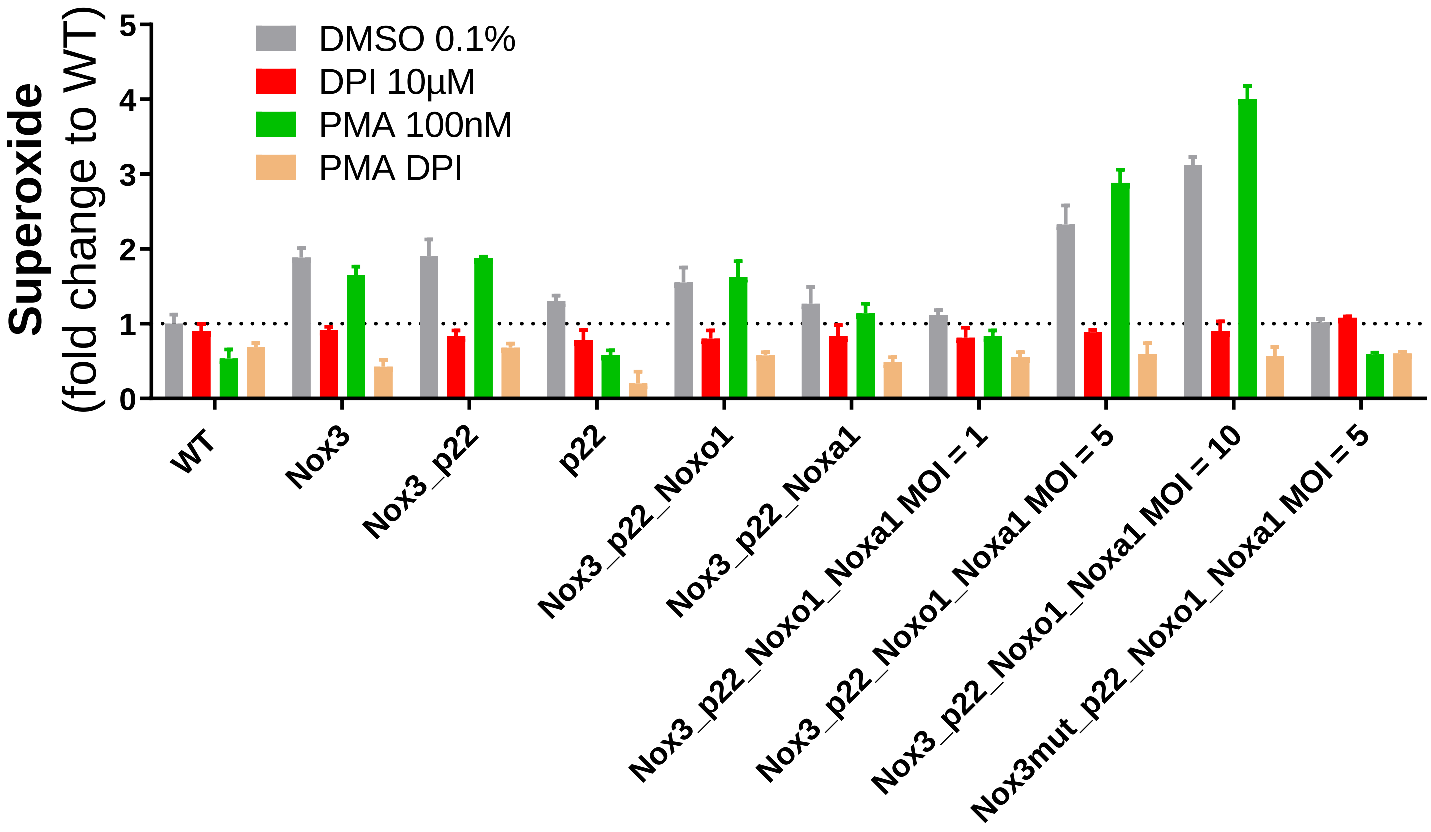

Supplementary Figure 1: Generation and characterization of NOX3 cell lines. (A) Each gene encoding for a subunit of the NOX3 complex was cloned under a selectable marker, NOX3-GFP, Cyba-mCherry, NOXO1-blasticidin and NOXA-puromycin in three cell lines, namely, HEK, HeLa and CHO. (B) Cells expressing all subunits of the NOX3 complex were selected upon antibiotics pressure (NOXO1 and NOXA1) and sorted by FACSAria II based on GFP and mCherry expression (NOX3 and Cyba). (C) Example of dot plot obtained from the cell sorting with FACSAria II. (D) different multiplicity of infection (MOI) were employed for cell transduction of NOX3 and its subunits Cyba, NoxO1 and NoxA1. In any case, the catalytic NOX3 subunit was limiting allowing for direct visualization of siRNA impact on NOX3 activity. NOX3 subunit expression was measured by qPCR and the table shows the Ct values of transduced subunits and the house keeping gene Gapdh. E) NADPH oxidase activity of NOX3 was measured in each combination of transduced CHO cells by using WST1 colorimetric assay. Cells were treated with DMSO, PMA (activator) and/or DPI (inhibitor). Data were normalized to WT control responses in each case. Abbreviations: DMSO, dimethyl sulfoxide; PMA, phorbol 12- myristate 13-acetate; DPI, diphenyliodonium. Data represent the average of three independent experiment +- SEM.

Sup. Figure 2

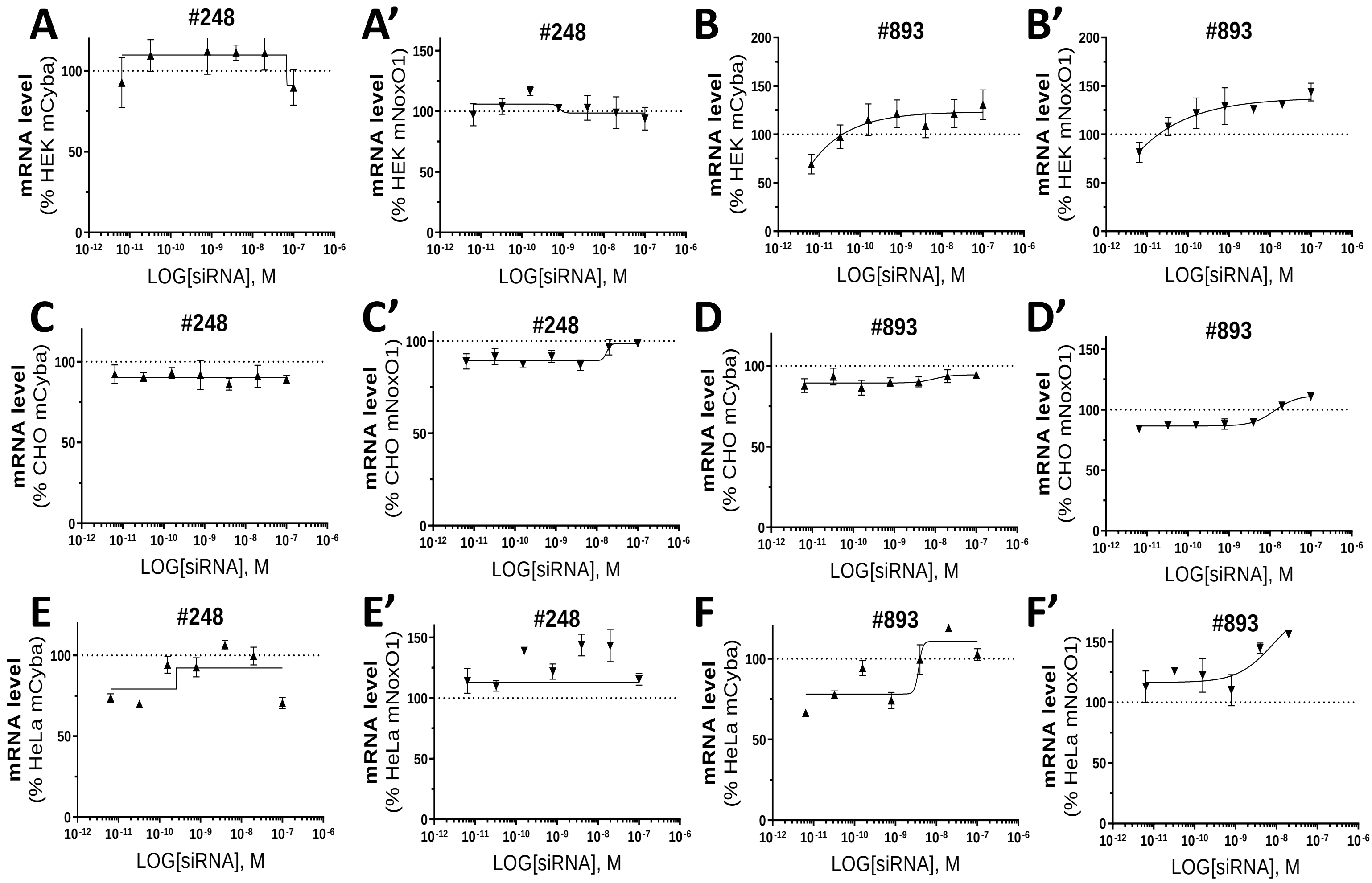

Supplementary Figure 2: Impact of siRNA #248 and #893 on NOX3 subunits *Cyba* and *NoxO1* in the three mNOX3 expressing cell lines. (A – F) *Cyba* and (A' – F') *NoxO1* mRNA as measured by qPCR in NOX3 expressing (A-B) HEK, (C-D) CHO and (E-F) HeLa transfected with increasing concentrations (100 nM – 0.0064 nM) of siRNA #248 (A, C, E) and #893 (B, D, F). siRNA concentrations are represented as the logarithm of the concentration (M). mRNA expression was normalized to non-transfected cells. Data represent the average of three independent experiment +-SEM.

Sup. Figure 3

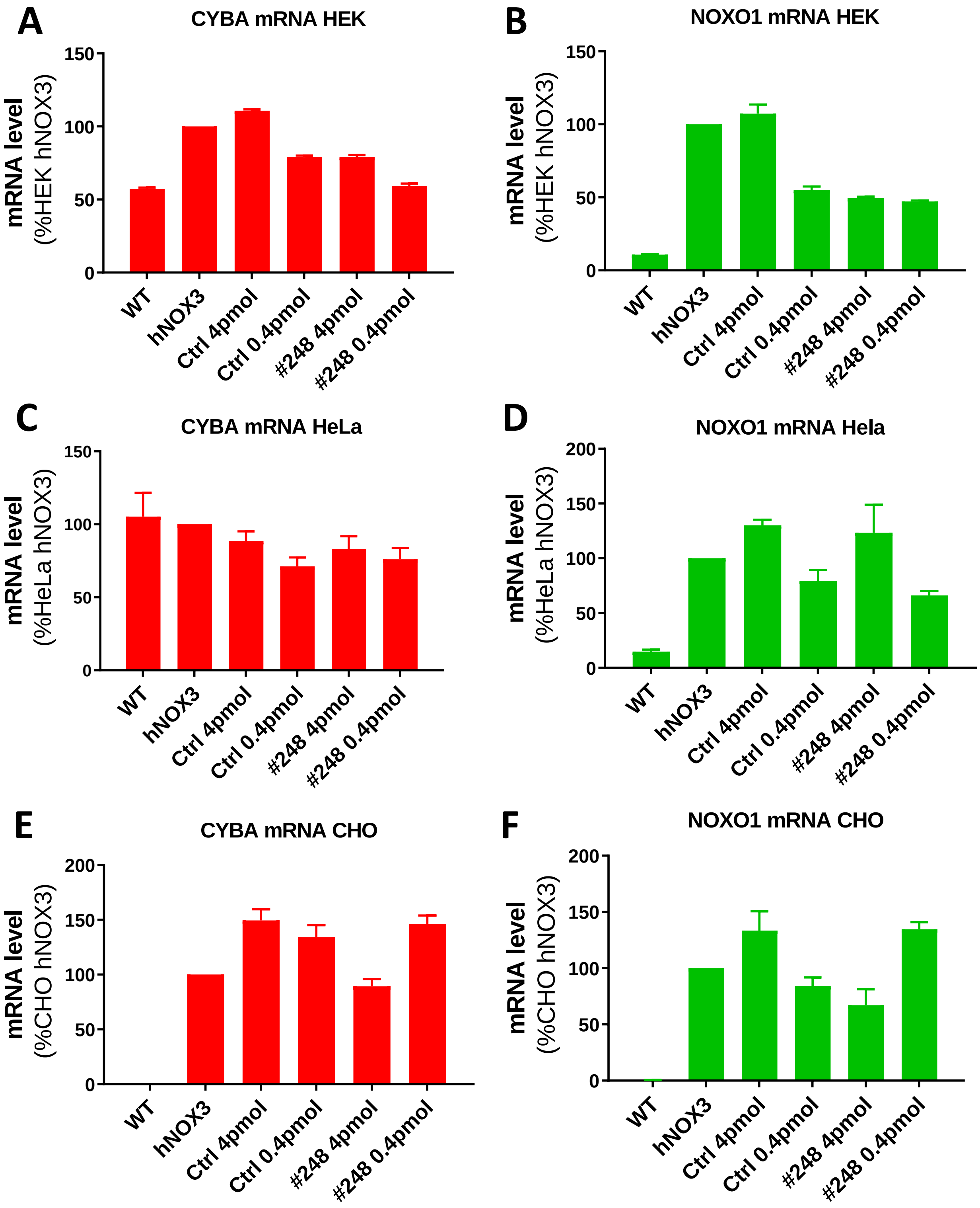

Supplementary Figure 3: Impact of siRNA #248 on other subunits of the human NOX3 complex. A, B) HEK, (C, D) HeLa and (E, F) CHO cell lines expressing the human NOX3 complex (NOX3, p22^{phox}, NOXO1 and NOXA1) were transfected with siRNA #248 or control siRNA (Ctrl) at two concentrations (4 and 0.4 pmol). 48h later, (A, C, E) *CYBA* and (B, D, F) *NOXO1* mRNA was measured by qPCR in the three cell lines. mRNA expression was normalized to non transfected hNOX3 expressing cells (100%). Data represent the average of three independent experiments +- SEM.

Sup. Figure 4

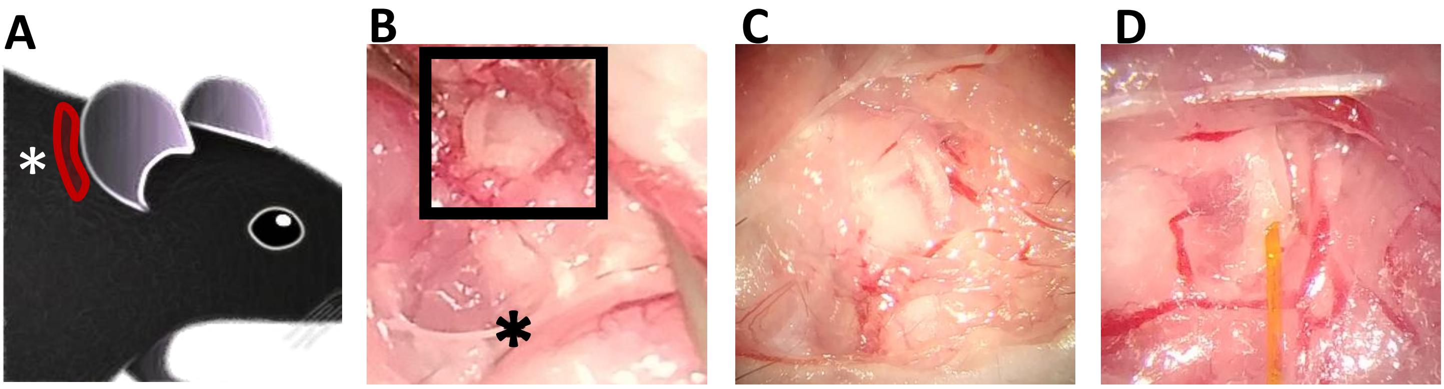

Supplementary Figure 4. siRNA delivery through posterior semicircular canalostomy. (A) In order to access the vestibular posterior canal, the mouse is placed on a lateral position, uncovering the right ear. An incision is made dorsally to the ear pinna midline, revealing the dorsal section of the cervical trapezius (B), located above the great auricular nerve (*). Following dissection, the posterior canal can be localized on the mouse skull surface (C). A 24 G needle was employed for drilling the bony cover, followed by cannulation (D). 1 µL of siRNA was manually delivered into the inner ear.

Sup. Figure 5

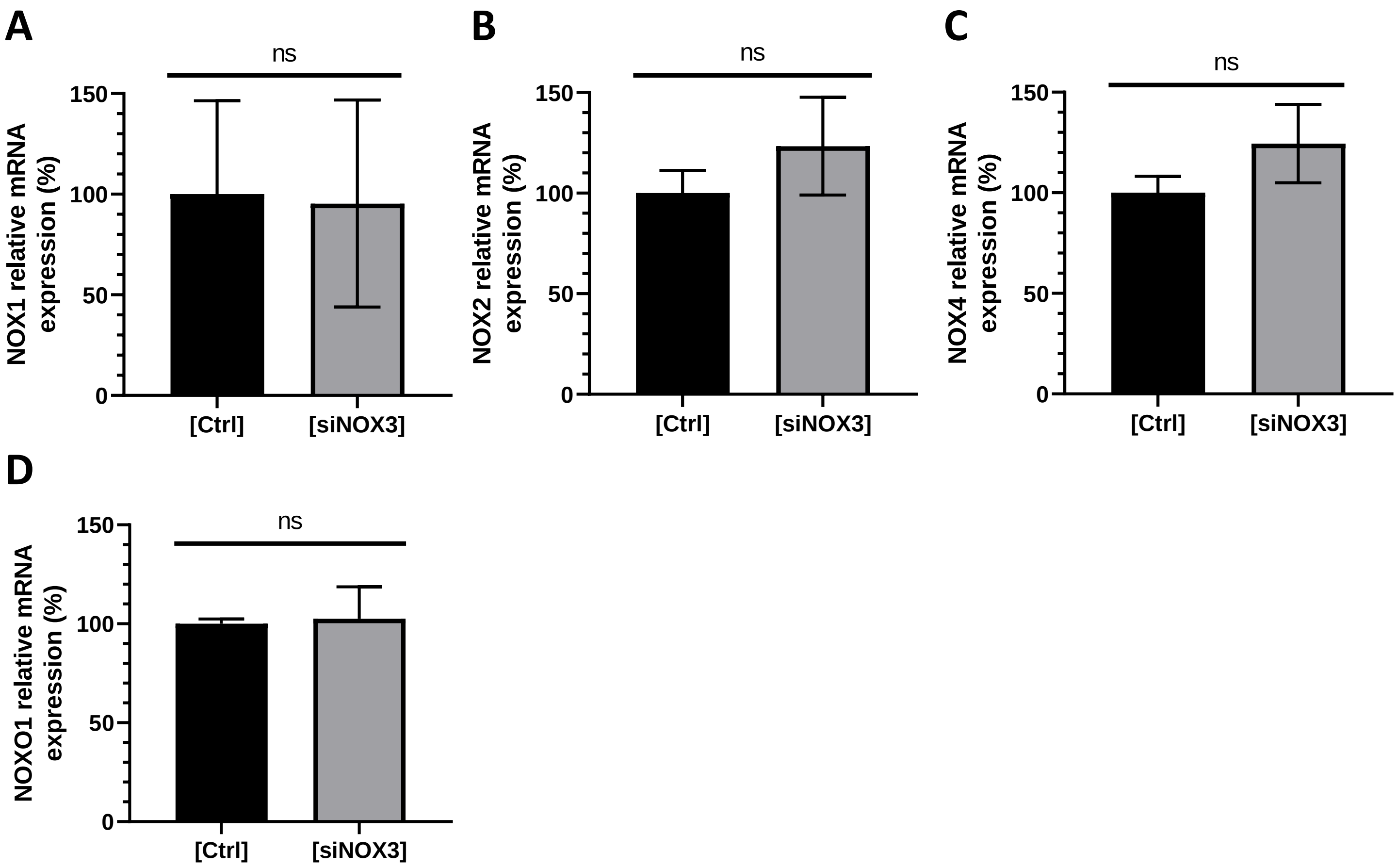

Supplementary Figure 5: Effect of siRNA #248 delivered through posterior semicircular canal on the mRNA level of *Nox1, 2, 4* and *NoxO1*. mRNA expression of *Nox1, 2, 4* and *NoxO1* were measured by real time qPCR 3 days following siNOX3 inner ear delivery. Quantitative comparison was performed between non-operated [Ctrl] and operated [siNOX3] ears. Data was normalized to the non-operated ear (%). (**A**) *Nox1* relative mRNA expression. (**B**) *Nox2* relative mRNA expression. (**C**) *Nox4* relative mRNA expression. (**D**) *NoxO1* relative mRNA expression. n=6 animals.

Sup. Table 1

Gene (Mm)	Primer forward 5'-3'	Primer reverse 5'-3'
Nox3	CGACGAATTCAAGCAGATTGC	AAGAGTCTTTGACATGGCTTTGG
Cyba	TGGACGTTTCACACAGTGGT	TGGACCCCTTTTTCCTCTTT
Noxa1	TTACTGTGCCCCTGAAGGTC	GGGGCTTTGTAACTGAGCTG
Noxo1	ATGCGGAAAACCCAGTACAC	TCGAAGCACTCACAATCCAG
Eef1A1	TCCACTTGGTCGCTTTGCT	CTTCTTGTCCACAGCTTTGATGA
Tubb	GCAGTGCGGCAACCAGAT	AGTGGGATCAATGCCATGCT
Actb	CTAAGGCCAACCGTGAAAAGAT	CACAGCCTGGATGGCTACGT

Gene (Hs)	Primer forward 5'-3'	Primer reverse 5'-3'
NOX3	ATGCCCCGTGCCTCAA	CCACAGGGCCTAAAATCCATT
CYBA	AAGAGGAAGAAGGGCTCCAC	CGGCCCGAACATAGTAATTC
NOXO1	GCACGAGCCGCGGCCTGGCG	GCTCCTCTGGGGTGGGCAGG
GAPDH	GCACAAGAGGAAGAGAGAGACC	AGGGGAGATTCAGTGTGGTG
B2M	TGCTCGCGCTACTCTCTCTTT	TCTGCTGGATGACGTGAGTAAAC
ACTB	GCACAGAGCCTCGCCTT	CCTTGCACATGCCGGAG

Gene (Cg)	Primer forward 5'-3'	Primer reverse 5'-3'
Actb	CGTGAAAAGATGACCCAGATCA	TTCTAGAGACAGCCGCATCTTTC
Gapdh	TTCTAGAGACAGCCGCATCTTTC	GCCGACCTTCACCATTGC
Tubb	GGGAAATCGTGCACATCCA	TGATCACCTCCCAGAACTTAGCA

Supplementary Table 1: List of qPCR primers employed in the study.
